# Overexpression of the Lipid Transfer Protein Gene *SpLTP1* from Desert Pioneer Plant *Stipagrostis pennata* Enhances the Drought Tolerance in *Arabidopsis*

**DOI:** 10.3390/plants14203198

**Published:** 2025-10-18

**Authors:** Jingru Wang, Jiahuan Niu, Ming Hu, Mingsu Chen, Xiaoying Li, Zhangqi Song, Shan Yin, Faren Zhu, Jiao Jiao, Rui Tang, Fei Wang, Rong Li, Hongbin Li

**Affiliations:** Key Laboratory of Oasis Town and Mountain-Basin System Ecology of Xinjiang Production and Construction Corps, Key Laboratory of Xinjiang Phytomedicine Resource and Utilization of Ministry of Education, College of Life Sciences, Shihezi University, Shihezi 832000, Chinaj2023j@126.com (J.J.); tr2604791944@163.com (R.T.);

**Keywords:** *Stipagrostis pennata*, lipid transfer protein, bioinformatics analysis, abiotic stress, subcellular localization, transcriptome analysis

## Abstract

Lipid transfer proteins (LTPs) play crucial regulatory roles in plant growth, development, and abiotic stress responses. *Stipagrostis pennata* is a species of grass widely distributed in arid and semi-arid regions, particularly adapted to desert and steppe environments. Under extreme drought conditions, it exhibits a variety of physiological and morphological adaptation mechanisms, making it an important species for studying plant drought tolerance. Recently, LTPs have been found to exhibit upregulated expression under drought stress in plants such as wheat and tobacco, enhancing their drought tolerance. However, the functional role of LTPs in *S. pennata* remains unexplored. In this study, the *SpLTP1* gene was isolated from *S. pennata* via molecular cloning, encoding a 116-amino acid protein. Phylogenetic analysis revealed that this protein contains a highly conserved nsLTP1 (cd01960) domain and has high sequence similarity with LTPs of *Setaria viridis*, *Setaria italica*, *Musa acuminata and Phragmites australis*. qRT-PCR revealed that *SpLTP1* was highly expressed and dynamically regulated under drought, suggesting its potential role in root rhizosheath formation and drought tolerance. To investigate *SpLTP1* function, *SpLTP1*-overexpressing (*SpLTP1-OE*) and complementation (*SpLTP1-atltp*) *Arabidopsis* lines were generated using the floral dip method, in comparison with the existing wild-type (WT) and the LTP-deficient mutant (*atltp*). Drought stress phenotyping and physiological assays indicated that *SpLTP1* likely enhances drought tolerance by elevating antioxidant enzyme activities and osmolyte accumulation. Comparative transcriptome analysis of *SpLTP1-OE* and WT plants further suggested that *SpLTP1* modulates critical pathways, including phenylpropanoid biosynthesis, zeatin biosynthesis, and plant hormone signal transduction, thereby influencing plant growth and stress adaptation. These findings not only provide novel insights into the molecular mechanisms by which *SpLTP1* regulates rhizosheath development in *S. pennata* but also establish a foundation for deciphering its role in extreme drought adaptation.

## 1. Introduction

Drought is an important environmental factor affecting plant growth and crop yield, and global climate change has aggravated the impact of drought stress on environment and crop production [1]. Xinjiang is a typical arid and semi-arid area, and it is the largest, most widely distributed and most seriously affected area of desertification land in China. Dry lands, whether influenced by natural conditions such as arid climates, sparse vegetation, and widespread deserts (including sandy deserts, gravel deserts, salt deserts, and rocky deserts) or by human activities, are highly susceptible to wind erosion and sediment accumulation, making them the most vulnerable areas to desertification [2]. Desert ecosystems harbor relatively few forage plant species, particularly among the *Poaceae* family [3]. The rhizosheath is an adaptive structure of higher plants in sandy environments. It is a soil continuum formed by mutual cementation and entanglement between soil particles, root surface secretions and root hairs, which can protect water and heat insulation, prevent physical damage from quicksand and improve nitrogen fixation efficiency of plants [4].

*Stipagrostis pennata* is a pioneer sand-fixing species in the desert in Xinjiang, China [5], which relies on its specialized rhizosheath as a structural foundation for drought tolerance [6]. The rhizosheath has a wind-proof and sand-fixing role in desert plants and plays an important role in desert adaptation [7]. It can protect the roots from the harsh environment, enhance the water absorption of the roots, and increase the exchange of information between the roots and the soil [8]. Under drought conditions, the rhizosheath can significantly improve the ability of the plant to withstand drought [9]. In agriculture, plants’ rhizosheath can also enhance the self-recovery of plants under drought stress [10]. Current studies suggest that carbohydrate and amino acid metabolism are closely linked to rhizosheath formation during drought [11,12]. The rhizosheath can prevent the root and soil from being separated due to root shrinkage, and the water retention capacity of the mucilage within the rhizosheath can further reduce water loss from the sheath to the non-rhizospheric soil [13,14,15,16,17]. Numerous plant species, including *Triticum aestivum*, *Zea mays*, and *Agropyron cristatum*, have been found to develop a rhizosheath. In recent years, limited research on these structures has primarily focused on their morphology, formation mechanisms, and ecological functions.

Lipid transfer proteins (LTPs) are small, alkaline proteins ubiquitous in plants, facilitating intercellular lipid transport and participating in cuticle synthesis and stress adaptation [18]. Under adverse stress conditions, plant cells initiate a series of responsive reactions, including physiological and metabolic alterations, changes in lipid composition, and hormone signal transduction [19]. Concurrently, stress tolerance-related genes are regulated to synthesize associated compounds, thereby maintaining ionic homeostasis within the plant. For instance, the LTPs of sugarcane respond to salicylic acid (SA) and methyl jasmonate (MeJA), and their expression was upregulated under cold and PEG treatment, which suggested their role in the occurrence of stress tolerance [20]. Under drought stress conditions, the expression levels of wheat *(T. aestivum*) LTPs are significantly upregulated. Water deficit leads to structural damage in the vascular bundle tissue layer, suggesting that wheat LTPs may play a protective role in maintaining cellular integrity of vascular bundle tissues under drought conditions [21]. Tobacco (*Nicotiana tabacum)* overexpressing NtLTPI.38 showed increased anti-oxidant capacity, reduced accumulation of radicals, increased fibrous roots and improved drought tolerance [22]. These results indicate the importance of LTPs in drought tolerance.

While prior studies on *S. pennata*, a xerophytic grass species of the *Poaceae* family, focused on population traits, ecological adaptation, and root microbiomes, the developmental mechanisms of rhizosheath formation remain unexplored. In our previous studies, we observed that *S. pennata* develops two distinct types of root systems under artificial cultivation: roots with a rhizosheath and non-rhizosheath roots. LTPs were found to be closely associated with rhizosheath development. Experiments revealed that *SpLTP1* has the highest expression in roots with a rhizosheath, suggesting its critical role in rhizosheath formation (unpublished experimental data). This study obtained the *SpLTP1* gene through molecular cloning, and the functional mechanism of *SpLTP1* in abiotic stress response and root development regulation was further studied, aiming to lay a foundation for better understanding the stress tolerance mechanism of *S. pennata*. The results of this study provide an important basis for further analysis of the adaptation of *S. pennata* to extreme desert environment. Meanwhile, the identification and functional study of the *SpLTP1* gene provide valuable genetic resources for plant stress tolerance gene engineering, and the elucidation of its molecular regulation mechanism can provide new theoretical guidance for crop stress tolerance improvement.

## 2. Results

### 2.1. Phylogenetic Analysis of SpLTP1 Gene Sequences Among Related Species

**Figure 1:**
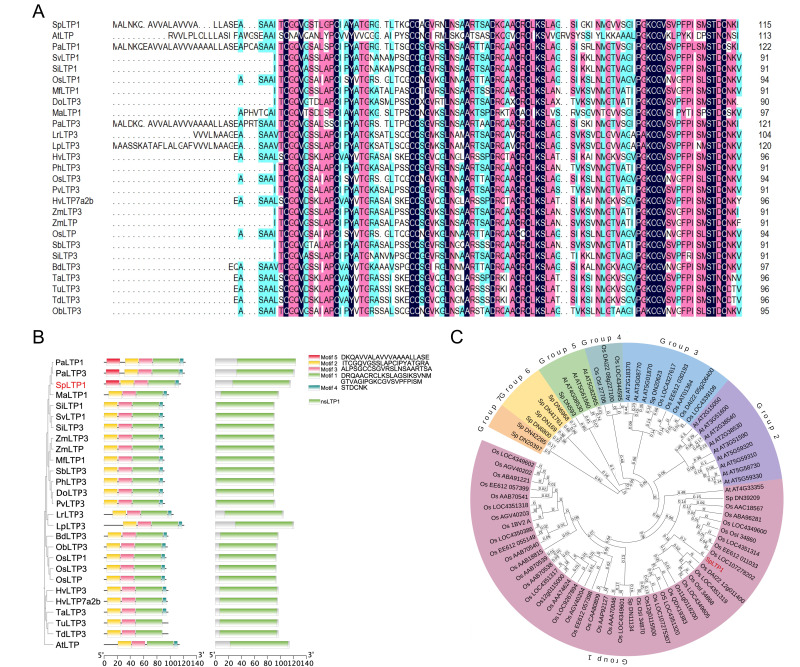
Sequence alignment, phylogenetic analysis, and conserved domain characterization of SpLTP1. (**A**) Multiple sequence alignment of SpLTP1 with orthologs from diverse species. The underlined region indicates the conserved nsLTP1 domain (cd01960). Color scheme for the multiple sequence alignment. Deep blue: denotes a completely conserved site (all sequences are identical); Light blue: denotes a partially conserved site (the majority of sequences are identical); Red: indicates that the site is matched in either 3 or 2 sequences; No color: denotes a site with significant variation. (**B**) Comprehensive domain architecture analysis of SpLTP1 and its homologs. Left panel: Phylogenetic relationships among species; Center: Distribution of characteristic motifs (Motif1–5); Right: Conserved domain organization.The red-colored region represents SpLTP1. (**C**) Maximum-likelihood phylogenetic tree of SpLTP1 with LTP family members from *O. sativa*, *Arabidopsis*, and *S. pennata*. Distinct clades are color-coded (Group 1–7); The red-colored region represents SpLTP1.

Through qRT-PCR analysis of 11 LTP genes in rhizosheath roots of *S. pennata* during normal growth at 90 days, we identified *SpLTP1* as the most highly expressed gene (Figure S1). Sequences of SpLTP1 protein were aligned by the NCBI blastp program, and 19 *Poaceae* species with high sequence similarity were selected, from which 25 homologous genes were identified. Additionally, AtLTP (*AT5G01870*) proteins exhibiting high similarity were screened from the *Arabidopsis* database via blastp for co-analysis (Table S1). The amino acid multiple sequence alignment results based on DNAMAN 9.0 software showed that the evolutionary relationships of these homologous genes were highly consistent with the botanical classification of their species, and all LTPs contained a common conserved domain nsLTP1 (cd01960), belonging to the AAI_LTSS superfamily. In the cross-species homology alignment, SpLTP1 has high sequence similarity with LTPs of *Setaria viridis, Setaria italica*, *Musa acuminata* and *Phragmites australis*. In addition, all LTPs contain three core motifs (Motif1, Motif2, and Motif3), while SpLTP1, PaLTP1 and PaLTP3 additionally contain Motif4, and LrLTP3 and LpLTP3 contain Motif5 (Figure 1A,B), further confirming the high conservation of LTPs. In order to explore the evolutionary relationship of LTP genes, phylogenetic trees were constructed for LTP gene family members in *Oryza sativa*, *Arabidopsis*, and *S. pennata*. The results showed that LTP genes in the three species were divided into seven distinct branches; SpLTP1 belonged to the Group 1 evolutionary branch and showed high homology with some LTP genes in *O. sativa* (Figure 1C).

### 2.2. Tissue-Specific Expression, Drought Response, and Subcellular Localization of SpLTP1

**Figure 2:**
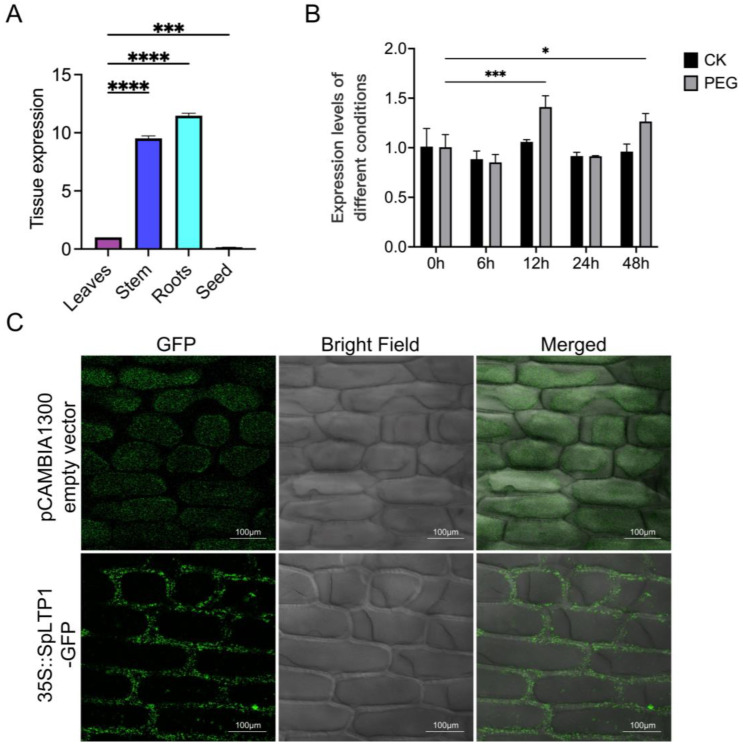
Expression profiling and subcellular localization analysis of *SpLTP1*. (**A**) Tissue-specific expression of *SpLTP1* in *S. pennata* leaves, stems, roots, and seeds. Asterisks denote statistical significance (vs. leaf tissues). (**B**) Expression levels of *SpLTP1* in *S. pennata* under control (CK) and drought (PEG) conditions at 0 h, 6 h, 12 h, 24 h, and 48 h. Asterisks denote statistical significance (vs. 0 h controls). (**C**) Subcellular localization of *SpLTP1-GFP* fusion protein in onion epidermal cells. Panels show: GFP signal (GFP), bright-field microscopy (Bright Field), and merged channels (Merged). Scale bar = 100 μm. Images represent observations under plasmolysis conditions. * *p* < 0.05, *** *p* < 0.001, **** *p* < 0.0001.

In order to investigate the expression level of *SpLTP1* in *S. pennata*, we collected samples from roots, stems, leaves, and seeds, as well as whole-plant materials subjected to drought stress for 0, 6, 12, 24, and 48 h. Quantitative real-time PCR (qRT-PCR) analysis revealed that *SpLTP1* was expressed across all examined tissues, with the highest expression in roots, followed by stem and leaves, and the lowest expression in seeds (Figure 2A). Under drought stress, *SpLTP1* expression decreased first and then increased, reaching a peak at 12 h of drought, then decreased at 24 h, and significantly increased at 48 h (Figure 2B). This tissue-specific expression pattern suggests that *SpLTP1* may play a key role in the development of the rhizosheath and may be involved in drought stress response in *S. pennata*. To determine the subcellular localization of *SpLTP1*, we constructed a *35S::SpLTP1-GFP* fusion vector and transiently expressed it in onion epidermal cells. Confocal microscopy confirmed cell wall localization of *35S::SpLTP1-GFP* (Figure 2C).

### 2.3. Involvement of SpLTP1 in Plant Drought Stress Response

**Figure 3:**
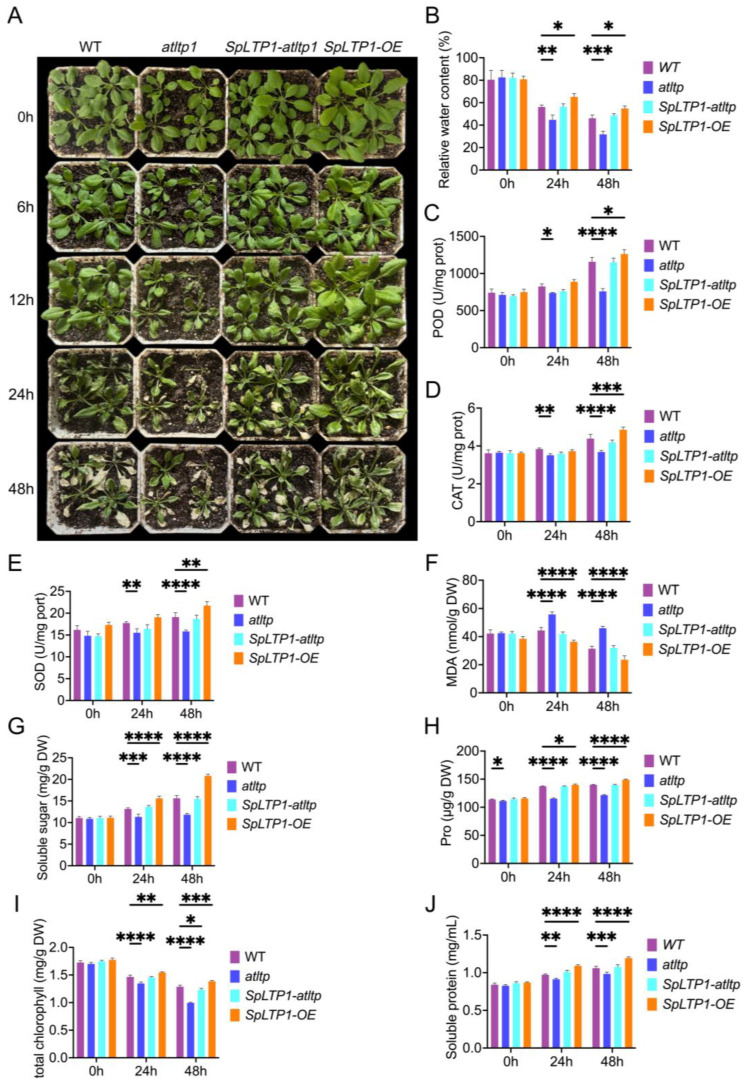
Phenotypic and physiological analyses of *Arabidopsis* under drought stress. (**A**) Growth phenotypes of *Arabidopsis* at 0, 6, 12, 24, and 48 h post-treatment (hpt) with 20% PEG. (**B**–**J**) Physiological parameter quantification: (**B**) Relative water content. (**C**) Peroxidase (POD) specific activity. (**D**) Catalase (CAT) specific activity. (**E**) Superoxide dismutase (SOD) specific activity. (**F**) Malondialdehyde (MDA) content. (**G**) Soluble sugar content. (**H**) Proline (Pro) content. (**I**) Total chlorophyll content. (**J**) Soluble protein content. Statistical significance (vs. 0 h controls) is indicated as: * *p* < 0.05, ** *p* < 0.01, *** *p* < 0.001, **** *p* < 0.0001.

To investigate plant responses to drought stress, we generated *Arabidopsis* overexpressing *SpLTP1* (*SpLTP1-OE*) and complemented mutant (*SpLTP1-atltp*) lines using the *35S::SpLTP1-GFP* vector. Four *Arabidopsis* genotypes (wild-type WT, *SpLTP1-OE*, mutant *atltp*, and complemented mutant *SpLTP1-atltp*) were subjected to 20% PEG6000 treatment for 0, 6, 12, 24, and 48 h. All lines exhibited varying degrees of leaf wilting and curling under stress (Figure 3A). After 6 h of treatment, *atltp* plants showed initial curling and wilting symptoms. By 24 h, all genotypes displayed leaf curling and withering, with *atltp* being the most severely affected and *SpLTP1-OE* showing minimal damage. After 48 h of drought treatment, the *SpLTP1-OE* transgenic plants exhibited a smaller decline in relative water content (RWC) and demonstrated stronger drought resistance compared to the other three genotypes (Figure 3A,B). Under normal growth conditions, no significant differences were observed in the physiological indices among the four *Arabidopsis* lines. However, under drought stress conditions, the *SpLTP1-OE* transgenic line exhibited significantly enhanced drought tolerant characteristics. Specifically, the transgenic line showed marked activation of its antioxidant enzyme system: the specific activities of peroxidase (POD) and catalase (CAT) were significantly elevated, peaking at 48 h (Figure 3C,D), while superoxide dismutase (SOD) specific activity ultimately increased by 0.14-fold compared to the WT (Figure 3E). Concurrently, membrane lipid peroxidation was substantially alleviated, and the Malondialdehyde (MDA) content in *SpLTP1-OE* plants was 25.0% lower than that in WT after 48 h of stress (Figure 3F). Regarding osmotic adjustment, *SpLTP1-OE* plants demonstrated continuous accumulation of soluble sugars and proline, reaching maximum levels at 48 h (Figure 3G,H). Furthermore, *SpLTP1* conferred protection to the photosynthetic system, reducing the chlorophyll content decline by 21.47% compared to 0 h under drought stress, significantly outperforming the *atltp* mutant (41.18%) (Figure 3I). Moreover, the soluble protein content in *SpLTP1-OE* significantly increased at both 24 h and 48 h (Figure 3J).

These results conclusively demonstrate that *SpLTP1* likely enhances *Arabidopsis* drought tolerance through coordinated mechanisms including strengthened antioxidant defense, improved osmotic adjustment, and reduced chlorophyll oxidative damage.

### 2.4. SpLTP1 Promotes Arabidopsis Growth Through Root Development

**Figure 4:**
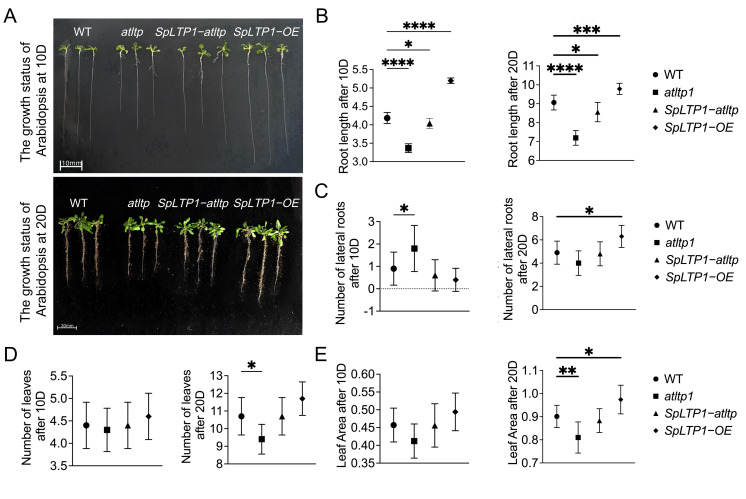
Statistical analysis of root length, lateral root number, leaf number, and leaf area in *Arabidopsis* at 10D and 20D. (**A**) Comparison of *Arabidopsis* growth status at 10D and 20D. (**B**) Quantification of primary root length at 10D and 20D; the unit of measurement is cm (centimeters). (**C**) Lateral root number enumeration at 10D and 20D. (**D**) Rosette leaf count at 10D and 20D. (**E**) Leaf area measurement at 10D and 20D; the unit of measurement is cm^2^ (square centimeters). Statistical significance (vs. WT): * *p* < 0.05, ** *p* < 0.01, *** *p* < 0.001, **** *p* < 0.0001.

In order to further understand the effect of *SpLTP1* on the growth and development of *Arabidopsis*, growth and development phenotypic analysis was performed on WT, *atltp*, *SpLTP1-OE*, and *SpLTP1-atltp* under standard culture conditions (Figure 4A). After 10 days of culture, root lengths of different genotypes showed significant differences, among which *SpLTP1-OE* grew fastest and *atltp* grew slowest. This growth difference was further amplified after 20 days of culture, indicating that *SpLTP1* gene has a continuous promotion effect on root development (Figure 4B). Statistical analysis of lateral root number revealed that *atltp* showed increased lateral root number density at 10D, but the number of lateral roots of *SpLTP1-OE* increased significantly at 20D (Figure 4C). Phenotypic analysis of leaf development showed that although there was no significant difference among the lines at 10D, and the rosette leaves increased in all genotypes at 20D, the increase was smaller in *atltp*, while no significant difference was found among the other genotypes (Figure 4D). Further measurements of *Arabidopsis* leaf area revealed an increase in all genotypes, with *SpLTP1-OE* exhibiting the greatest expansion, while *atltp* showed the smallest increase (Figure 4E). The results collectively demonstrate that the *SpLTP1* gene plays a crucial role in promoting *Arabidopsis* growth and development. *SpLTP1* regulates overall plant growth by modulating root system development and leaf expansion.

### 2.5. SpLTP1 Promotes Arabidopsis Development by Activating Phenylpropanoid Biosynthesis Pathways and Zeatin Biosynthesis Pathways

**Figure 5:**
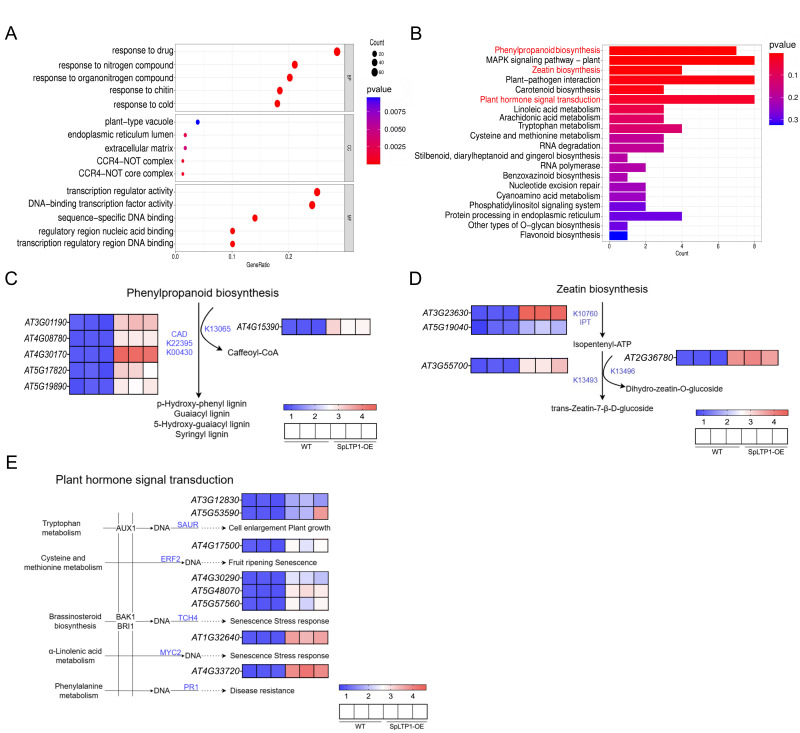
KEGG and GO analysis of upregulated genes in the *SpLTP1* transcriptome. (**A**) GO enrichment analysis of upregulated genes in *SpLTP1-OE* vs. WT. (**B**) KEGG pathway enrichment analysis of upregulated genes in *SpLTP1-OE* vs. WT. The red area in the figure corresponds to the KEGG pathway depicted in (**C**–**E**). (**C**) Phenylpropanoid biosynthesis pathway and gene expression levels of upregulated genes in *SpLTP1-OE* vs. WT. (**D**) Zeatin biosynthesis pathway and gene expression levels of upregulated genes in *SpLTP1-OE* vs. WT. (**E**) Plant hormone signal transduction pathway and gene expression levels of upregulated genes in *SpLTP1-OE* vs. WT. The color bars in the lower right corner of (**C**–**E**) represent the relative gene expression levels, with the scale indicating the material names. Each material group includes three biological replicates.

To elucidate the molecular regulatory mechanisms underlying the effects of *SpLTP1* overexpression in *Arabidopsis,* we performed RNA-seq analysis on whole-plant materials of *SpLTP1-OE* and WT *Arabidopsis* and conducted systematic functional annotation analysis of 369 differentially expressed genes (DEGs) significantly upregulated in *SpLTP1-OE* versus WT comparison groups (Figure S2). GO enrichment analysis results showed that these differential genes were significantly enriched at multiple functional levels: the most significantly enriched pathways in biological processes (BP) included response to drug (GO:0042493), response to nitrogen compound (GO:1901698) and response to organic nitrogen compound (GO:0010243); in molecular functions (MF), they were significantly enriched in transcription regulator activity (GO:0140110), DNA-binding transcription factor activity (GO:0003700) and sequence-specific DNA binding (GO:0043565) (Figure 5A). KEGG pathway analysis further revealed that these DEGs were extensively involved in several important metabolic and signaling pathways (Figure 5B). Phenylpropanoid biosynthesis (ko00940) was the most abundant pathway, followed by plant MAPK signaling pathway (ko04016) and Zeatin biosynthesis (ko00908).

Through in-depth analysis of KEGG metabolic pathway, this study revealed several key metabolic networks regulated by *SpLTP1* gene (Figure 5C–E), and the expression levels were further validated by qRT-PCR, and the validation confirmed that the expression trends of the selected genes were highly consistent with the transcriptomic data. In the Phenylpropanoid biosynthesis pathway, we identified five key genes (*AT3G01190/AT4G08780/AT4G30170/AT5G17820/AT5G19890*) that were significantly upregulated via the CAD (Cinnamyl Alcohol Dehydrogenase Pathway, K22395/K00430) pathway. These genes promoted the formation of four major lignin monomers (p-hydroxyphenyl lignin, guaiacyl lignin, 5-hydroxyguaiacyl lignin, and syringyl lignin), and *AT4G15390* (K13065) was upregulated in caffeoyl-CoA biosynthesis (Figure 5C). In the zeatin biosynthesis pathway, the *AT3G1530* and *AT5G19040* genes participate in the synthesis of Isopentenyl-ATP via the IPT (Isopentenyltransferase Pathway, K0760). Meanwhile, *AT2G36780* (K3496) regulates the production of Dihydrozeatin-O-glucoside, and *AT3G55700* (K13493) is involved in the synthesis of trans-Zeatin-7-β-D-glucoside (Figure 5D). Plant hormone signal transduction analysis showed that differentially expressed genes were significantly enriched at several key metabolic nodes, including tryptophan metabolism, cysteine and methionine metabolism, brassinosteroid biosynthesis, α-linolenic acid metabolism and phenylalanine metabolism (Figure 5E). These findings suggest that *SpLTP1* may regulate plant growth and development by coordinating the synthesis and signal transduction of multiple plant hormones.

## 3. Discussion

This study initially revealed that the *SpLTP1* gene plays an important role in drought stress tolerance and may have an important impact on the development of the rhizosheath of *S. pennata*. It may help *S. pennata* adapt to the extreme arid desert environment by influencing its metabolic pathways, enhancing antioxidant defense mechanisms, improving cellular osmotic regulation, activating the SOD/POD system, scavenging ROS (reactive oxygen species), promoting proline expression, maintaining cell membrane stability, and reducing oxidative damage [23,24]. These findings provide new clues for in-depth analysis of the adaptive mechanism of desert plants, and future studies could further focus on the dynamic expression patterns of this gene under drought stress and its regulatory network.

This study focused on the *SpLTP1* gene of *S. pennata* and made important discoveries at several key levels, which provided valuable information for further elucidating the mechanism of this gene in plant drought tolerance, particularly in *S. pennata*’s drought adaptation. *SpLTP1* gene encodes a protein of 116 amino acids and possesses a highly conserved nsLTP1 domain (cd01960). The existence of this conserved domain suggests that *SpLTP1* may perform similar basic biological functions in different plant groups, and the close relationship with *S. viridis*, *S. italica*, *M. acuminata* and *P. australis* provides important clues for subsequent cross-species comparison studies, which is helpful to explore the conservation and differentiation of this gene function from a wider plant category. *SpLTP1* and some *O. sativa* LTPs clustered in Group 1, suggesting that *SpLTP1* may share functional similarities with *O. sativa* homologous genes, such as strengthening the plant structural barrier and protecting organs against mechanical disruption and pathogen attack [25]. The qRT-PCR results revealed that the *SpLTP1* gene is highly expressed in the roots of *S. pennata* and plays a crucial role in its drought tolerance. This finding provides a crucial entry point for further investigation into the molecular mechanisms of rhizosheath formation and drought tolerance in *S. pennata*, suggesting that *SpLTP1* may act as an important regulatory factor in this process.

Subcellular localization analysis revealed that the *SpLTP1-GFP* signal was predominantly localized in the cell wall region, consistent with the secretory nature of LTPs [26]. This cell wall localization suggests that *SpLTP1* may function in cuticle formation and reinforcement of physical barriers against drought and pathogens. Furthermore, physiological assays in *Arabidopsis* under drought stress conditions confirmed the critical role of *SpLTP1* in plant drought response. These findings align with recent studies on the involvement of plant LTPs in abiotic stress responses [27], highlighting the gene’s importance in stress adaptation and suggesting a potential intrinsic link between rhizosheath development and drought tolerance mechanisms—possibly an evolutionary strategy for survival in arid sandy environments. Based on existing research, we speculate that *SpLTP1* may play a potential role in plant cell wall formation and growth regulation [28]. Additionally, lateral root development is likely finely modulated by phytohormones such as auxin and cytokinin [29]. LTPs may influence leaf expansion by affecting cuticular lipid deposition or cell wall loosening [30], suggesting that *SpLTP1* could regulate cell wall plasticity or photosynthetic efficiency.

Transcriptome analysis provided further compelling evidence for elucidating the molecular mechanisms of *SpLTP1*. The study revealed that *SpLTP1* likely enhances the survival ability of *S. pennata* in arid environments through multiple metabolic pathways. GO enrichment analysis showed that upregulated DEGs were significantly enriched in drug response and nitrogen compound metabolic pathways. The drug response pathway in plants is often associated with stress responses, where external stresses such as drought may be perceived as drug-like stimuli, activating a series of defense mechanisms through this pathway [31]. The involvement of *SpLTP1* in this process suggests its critical role in stress signal transduction and response. Meanwhile, the nitrogen compound response pathway may influence nitrogen utilization and metabolism under stress conditions, maintaining essential physiological functions, which is closely linked to plant survival strategies under drought [32]. KEGG enrichment analysis demonstrated that *SpLTP1* overexpression affected key metabolic pathways, including phenylpropanoid biosynthesis, zeatin biosynthesis, and plant hormone signal transduction. Phenylpropanoid-derived compounds, such as lignin, play a crucial role in cell wall structure and function [33]. The rhizosheath is a specialized cell wall structure, and we hypothesize that *SpLTP1* may function in the biosynthesis of plant cell wall components (e.g., cutin monomers) and indirectly influence the deposition of phenylpropanoid-derived polymers (such as lignin and cutin) through the phenylpropanoid biosynthesis pathway [34,35]. Zeatin, a major cytokinin (CK), regulates *Arabidopsis* root development by influencing taproot formation and primary root elongation through signal transduction, balancing auxin distribution [36]. Meanwhile, the plant activates its defense responses by participating in the signaling transduction of hormones such as ABA and JA, while enhancing their interactions with cell membrane receptors [37,38]. These findings indicate that *SpLTP1* may influence plant growth and stress adaptation by regulating secondary metabolism, hormone homeostasis, and stress responses. In this study, we primarily analyzed the upregulated genes (UDEGs) within the transcriptome data. To fully decipher the complex mechanisms involved, future work will focus on analyzing the regulatory networks potentially centered on these significantly downregulated genes. This study provides novel insights into the molecular mechanisms of *SpLTP1* in plant stress adaptation and lays a foundation for exploring its downstream regulatory network.

However, this study still leaves several questions open for further investigation. Although we have identified several pathways associated with rhizosheath development and drought stress response in which *SpLTP1* participates, the synergistic relationships among these pathways and the precise regulatory nodes occupied by *SpLTP1* within these complex networks demand more in-depth exploration. Additionally, although whole-plant transcriptome analysis may be influenced by organ proportions, this study minimized such bias through rigorous phenotypic matching and proportional normalization. Future investigations may employ organ-specific transcriptomics for further analysis. Furthermore, since this study primarily employed *Arabidopsis* as the model system, future research should include functional validation experiments in the native *S. pennata* to ensure the applicability and accuracy of the findings in natural ecological contexts. In summary, through multidimensional analyses, this study has preliminarily revealed the crucial role and potential mechanisms of the *SpLTP1* gene in rhizosheath development and drought stress response in *S. pennata*, laying a solid foundation for further investigation into its functional characterization and the molecular basis of desert adaptation. Nevertheless, subsequent studies addressing the aforementioned aspects are essential to comprehensively elucidate the biological functions and mechanistic details of the *SpLTP1* gene.

## 4. Materials and Methods

### 4.1. Plant Materials

The experimental materials were derived from mature seeds of *S. pennata* collected annually in June through bagging techniques in the desert adjacent to Mosuowan Reservoir, Shihezi City, Xinjiang Uygur Autonomous Region. The seeds were decorticated, pretreated with a 0.03% gibberellin (GA_3_) solution, and subsequently sown in sterilized sand. Cultivation was conducted under constant temperature conditions (32 °C) to obtain intact plants for further experimental investigations.

### 4.2. Instruments and Reagents

The total RNA extraction kit (DP432), first-strand cDNA synthesis kit (KR116), DNA gel recovery kit (DP214), and plasmid extraction mini kit (DP103) were purchased from TIANGEN Biotech (Beijing, China). 2×Taq PCR Master Mix II (P132) and Real-time PCR reagents were purchased (Q312) from Vazyme Biotech (Nanjing, China). The pMD-19 T cloning vector was obtained from TaKaRa Bio (Shiga, Japan). T4 DNA Ligase (2011A) was supplied by TaKaRa Bio (Shiga, Japan). Restriction enzymes (*Kpn*I, *Bam*HI, etc.) were purchased from TaKaRa Bio (Shiga, Japan). Kanamycin, gentamicin, MES (2-(N-morpholino) ethanesulfonic acid), acetosyringone, MgCl_2_, and other medium components (analytical grade) were procured from Sangon Biotech (Shanghai, China). The *Agrobacterium tumefaciens* strain GV3101 and the subcellular localization vector pCAMBIA1300 were preserved at the Key Laboratory of Agricultural Biotechnology, College of Life Sciences, Shihezi University (Xinjiang, China). The *Escherichia coli* competent cells (DH5α) were purchased from TransGen Biotech (Beijing, China). PCR primer synthesis and DNA sequencing were performed by Youkang Biotechnology Co., Ltd. (Xinjiang, China). *Arabidopsis* mutant seeds were obtained from AraShare Scientific (www.arashare.cn, accessed on 1 March 2025). The following biochemical assay kits were acquired from Solarbio (Beijing, China): Peroxidase (POD) Activity Assay Kit (BC0095), Superoxide Dismutase (SOD) Activity Assay Kit (BC0175), Malondialdehyde (MDA) Content Assay Kit (BC0025), Catalase (CAT) Activity Assay Kit (BC4785), Plant Soluble Sugar Content Assay Kit (BC0035), Proline (Pro) Content Assay Kit (BC0295), and Plant Chlorophyll Content Assay Kit (BC0995).

### 4.3. Construction of Overexpression Vector and Identification of Transgenic Plants

#### 4.3.1. Treatment of *S. pennata*

*S. pennata* plants were collected after 45D of growth under normal conditions, with roots, stems, leaves and seeds collected from 10 whole plants each for subsequent use. Additionally, whole plants treated with 20% PEG solution for 0 h, 6 h, 12 h, 24 h, and 48 h were collected (15 plants per time point), with each treatment group divided into three biological replicates for further analysis.

#### 4.3.2. RNA Extraction and cDNA Synthesis of *S. pennata*

Total RNA was extracted from the following samples using the RNA extraction kit according to the manufacturer’s instructions: whole-plant materials, roots, stems, leaves, and seeds of *S. pennata*, and root materials with rhizosheath from *S. pennata* grown normally for 90 days, as well as whole-plant materials subjected to drought stress for 0 h, 6 h, 12 h, 24 h, and 48 h. RNA integrity was verified by 1.2% agarose gel electrophoresis, and concentration was measured using a spectrophotometer (NanoDrop 2000/One, Thermo Fisher, Waltham, MA, USA). First-strand cDNA was synthesized from total RNA using a reverse transcription kit and stored at −20 °C for downstream applications.

#### 4.3.3. Cloning of *SpLTP1* Gene

The coding sequence (CDS) of *SpLTP1* was obtained from the transcriptome data of *S. pennata*. Gene-specific primers (SpLTP1-F and SpLTP1-R) and restriction site-containing primers (SpLTP1-kb-F and SpLTP1-kb-R; see Table 1) with *Kpn*I and *Bam*HI sites were designed using Premier 5.0. PCR amplification was performed using cDNA from sterile-grown *S. pennata* as a template with 2× Taq PCR Master Mix. The 20 μL PCR reaction mixture contained: 1 μL cDNA (200 ng/μL), 1 μL each of forward (SpLTP1-F) and reverse (SpLTP1-R) primers, 10 μL 2× Taq PCR Master Mix, and 7 μL ddH_2_O. PCR conditions: Initial denaturation: 95 °C for 3 min. 35 cycles of: Denaturation: 95 °C for 30 s; Annealing: 60 °C for 30 s; and Extension: 72 °C for 1 min. Final extension: 72 °C for 5 min. The amplified product was electrophoresed on a 1.2% agarose gel, and the target band was purified using a gel extraction kit. The purified fragment was sequenced for verification.

#### 4.3.4. Sequence Analysis of the Gene

Sequence alignment was performed using DNAMAN (Version 9). The conserved domain of SpLTP1 from *S. pennata* was analyzed using the NCBI Conserved Domains online. Phylogenetic analysis was performed using MEGA (Version 11) [39] and Itol (https://itol.embl.de accessed on 10 March 2025 ), while motif analysis was carried out using the MEME online suite [40]. Visualization of phylogenetic relationships, conserved domains, and motifs was generated using TBtools (v2.210) [41].

#### 4.3.5. Construction of Plant Expression Vector

The verified *SpLTP1* sequence was ligated into the pMD-19T vector and transformed into *E. coli* DH5α competent cells. Positive clones were selected on LB solid medium containing ampicillin (100 μg·mL^−1^) after 12 h of incubation and sent to Sangon Biotech (Shanghai, China) for sequencing validation. Sequence alignment was performed using DNAMAN, and correctly aligned clones were cultured for plasmid extraction using a commercial plasmid extraction kit. The pCAMBIA1300-GFP vector (containing the 35S promoter) and the verified pMD-19T-*SpLTP1* plasmid were double-digested with *Kpn*I and *Bam*HI. The linearized *SpLTP1* fragment and vector were gel-purified and ligated using T4 DNA ligase. The recombinant plasmid was transformed into *E. coli* DH5α and plated on LB agar with ampicillin (100 μg·mL^−1^). After 12 h incubation at 37 °C, positive colonies were screened by colony PCR and verified by sequencing (Sangon Biotech, Shanghai, China) to obtained *35S::SpLTP1-GFP* (Figure S1). The correct *35S::SpLTP1-GFP* recombinant plasmid was introduced into *A. tumefaciens* GV3101 via the freeze–thaw method [42]. Transformed cells were plated on LB medium containing triple antibiotics (gentamicin, 50 μg·mL^−1^; kanamycin, 50 μg·mL^−1^; rifampicin, 50 μg·mL^−1^) and incubated at 28 °C for 36 h. Positive colonies were confirmed by colony PCR, and validated strains were preserved for subsequent experiments.

#### 4.3.6. Subcellular Localization

Fresh onions were purchased and their roots were immersed in water until elongation. The outer three layers of scales were removed, followed by surface sterilization with 75% ethanol for 10 min in a laminar flow hood, and then rinsed three times with sterile water. Inner thick scales were selected and cut into 1 cm^2^ pieces, with the adaxial surface (facing mesophyll) placed on MS medium for dark incubation for 2 days. Recombinant *A. tumefaciens* harboring the target plasmid was activated in LB liquid medium supplemented with gentamicin (50 μg·mL^−1^), rifampicin (50 μg·mL^−1^), and kanamycin sulfate (50 μg·mL^−1^) at 28 °C with 100× g shaking for 12 h. Bacterial cells were collected by centrifugation at 3000× *g* for 10 min. Pre-cultured onion epidermal peels were immersed in bacterial suspension (OD_600_ = 1.0) containing MS liquid medium with 100 mmol·L^−1^ MES, 10 mmol·L^−1^ MgCl_2_, and 100 μmol·L^−1^ acetosyringone, followed by incubation at 28 °C with 100× g shaking for 30 min. After blotting with sterile filter paper, samples were placed adaxial side up on MS solid medium with filter paper and incubated in darkness for 3 days. Prior to observation, the samples were rinsed with ddH_2_O to remove residual bacteria. Subsequently, they were treated with 0.8 M sucrose solution to induce plasmolysis in the onion epidermal cells. Finally, observation and image acquisition were performed using a confocal laser scanning microscope. The empty vector control was pCAMBIA1300-GFP (without insert fragment), with confocal microscopy performed using a Zeiss LSM 980 (Carl Zeiss AG, Jena, Germany), Objective lens: Plan-Apochromat 63×/1.40 Oil DIC. Laser: Argon 488 nm, DPSS 561 nm, Airyscan 2 mode, GFP: BP488 nm.

#### 4.3.7. Gene Expression Analysis

Total RNA was extracted from treated *S. pennata* whole-plant materials, and root, stem, leaf, and seed materials and reverse-transcribed into cDNA. Gene-specific primers for *SpLTP1* were designed using Premier 5.0 (Table 1), with *GAPDH* [43] as the reference gene. qPCR was performed using 2× SuperReal PreMix (SYBR Green) on a Roche LightCycler 480 system with the following 10 μL reaction: 5 μL 2× SuperReal PreMix Plus, 0.2 μL each forward/reverse primer, 2.5 μL cDNA template, and 2.1 μL RNase-free ddH_2_O. The thermal profile included: 95 °C for 15 min (initial denaturation), followed by 40 cycles of 95 °C for 10 s, 52 °C for 20 s, and 72 °C for 20 s. Three technical replicates were performed per sample, and relative expression levels were calculated using the 2^−ΔΔCt^ method [44].

### 4.4. Phenotypic Observation and Physiological Index Analysis in Arabidopsis

The *Arabidopsis* homolog *AT5G01870* of *SpLTP1* was identified by blasting the *SpLTP1* nucleotide sequence against the *Arabidopsis* database. The T-DNA insertion mutant *atltp* (*AT5G01870,* SALK_060963C) was obtained from AraShare Scientific and cultivated alongside wild-type (WT) *Arabidopsis*.

Transgenic *Arabidopsis* lines were generated as follows: *SpLTP1*-overexpressing plants (*SpLTP1-OE*) were produced by floral dip transformation of WT *Arabidopsis* [45]; complementation lines (*SpLTP1-atltp*) were created by transforming the *SpLTP1* gene into the *atltp* mutant background. Genomic DNA was extracted from the plants, and PCR was performed using the primers SpLTP1-F and SpLTP1-R (method as described in Section 4.3.3) to verify the successful transformation of the *SpLTP1* gene (Figure S1B). The expression levels of endogenous *AtLTP* (primers in Table S3) and of *SpLTP1* (primers in Table 1) were measured in *Arabidopsis* lines to validate experimental accuracy (Figure S3).

For drought stress treatment, four genotypes (WT, *atltp*, *SpLTP1-atltp*, and *SpLTP1-OE*) at approximately 4 weeks old were irrigated with 20% PEG6000 solution (solution Ψ ≈ −0.5 MPa, moderate stress). Phenotypic analysis was performed using three independent T3-generation homozygous lines for each transgenic genotype. The method for determining relative water content is as follows: Take 0.2 g of leaves from four genotypes of *Arabidopsis* at 0 h, 24 h, and 48 h, denoted as W_f_ (fresh weight). Then, immerse the leaves in water for 8 h and weigh them to obtain the turgid weight (W_t_). After that, place the leaves in an oven at 105 °C for 15 min to deactivate enzymes, and then dry them at 75 °C until a constant weight is achieved. The dry weight is recorded as W_d_. The relative water content is calculated using the formula RWC (%) = (W_f_ − W_d_)/(W_t_ − W_d_) × 100 [46]. At 0 h, 24 h, and 48 h of PEG treatment, 15 whole plants and 9 leaf samples were collected from each of the four *Arabidopsis* lines, with each treatment group divided into three biological replicates, and oven-dried at 75 °C until constant weight to remove moisture and obtain the dry weight (DW). Subsequently, the whole-plant samples were analyzed for POD, CAT, SOD, MDA, PRO, and soluble sugar contents using the following Solarbio assay kits: POD Activity Assay Kit (BC0095), CAT Activity Assay Kit (BC4785), SOD Activity Assay Kit (BC0175), MDA Content Assay Kit (BC0025), Proline Content Assay Kit (BC0295), Soluble Sugar Content Assay Kit (BC0035) and Jingmei Plant Soluble Protein (S-protein) ELISA Kit (JM-110029P2). Meanwhile, chlorophyll content in the leaf samples was determined using the Solarbio Plant Chlorophyll Content Assay Kit (BC0995); all contents were measured based on plant DW, and enzyme activities were expressed as specific activity. All experimental procedures, reagents, and calculation methods strictly followed the manufacturer’s instructions provided with each kit. Graphical representations and analysis of significant differences were performed using two-way ANOVA with Dunnett’s multiple comparisons test in GraphPad Prism 10 [47].

### 4.5. Transcriptome Analysis of SpLTP1-Related Pathways in Arabidopsis

By collecting whole-plant materials of *SpLTP1-OE* and WT *Arabidopsis* under normal growth conditions at 20D, all samples were collected from plants at strictly synchronized growth stages, with sampling conducted only when the shoot/root fresh weight ratio showed no significant variation. Each biological replicate for RNA-seq was derived from a pooled sample of 10 uniformly grown plants. Sample were flash-frozen with liquid nitrogen and transported on dry ice to Wuhan IGENEBOOK Biotechnology Co., Ltd. in Wuhan, China, for RNA-seq detection. The raw image data files obtained from high-throughput sequencing using the Illumina HiSeq™ 2000 platform [48] were converted to raw reads through base calling. Quality control of the data was conducted using the fastqc software (version: 0.11.5) [49], and statistical analysis of the raw data and filtered high-quality data (clean reads) was performed to obtain basic data information (Table S2). The Clean Reads were aligned to the *Arabidopsis* genome using the hisat2 software (version: 2.0.1-beta) [50] to obtain valid reads. To compare gene expression differences between samples, differential expression analysis was performed using the R package edgeR (3.38.1) [51]. Genes with an FDR value less than 0.05 and an absolute fold-change greater than 2 were considered significantly differentially expressed genes (Figure S2). Subsequent transcriptomic GO and KEGG data analyses were carried out through the IGENEBOOK cloud platform (https://xcx.igenebook.cn, accessed on 7 May 2025). qRT-PCR was performed using the same *Arabidopsis* materials as those in the transcriptome analysis (method details as described in Section 4.3.7, the primer sequences are provided in Table S3) to validate the relative gene expression levels. The fold-change in expression levels was normalized using the mean value of three biological replicates in the WT group as the baseline.

## 5. Conclusions

This study successfully identified the *SpLTP1* gene, a lipid transfer protein from *S. pennata* localized to the cell wall. By investigating *SpLTP1* in its native species and after introducing it into the model plant *Arabidopsis*, we examined its role in drought stress response and normal growth and development through phenotypic characterization and RNA-seq analysis. The results indicate that *SpLTP1* likely enhances plant drought tolerance by boosting the activity of antioxidant enzymes and increasing osmotic adjustment substances. Furthermore, *SpLTP1* may influence plant growth, development, and stress responses by regulating key pathways such as phenylpropanoid biosynthesis, zeatin biosynthesis, and plant hormone signal transduction. This research provides a reference for understanding the function and regulatory mechanisms of LTP genes in *S. pennata*.

## Figures and Tables

**Table 1:** Primers used in this study.

Peimer	Primer Sequence (From 5′ to 3′)	Primer Function
SpLTP1-F	ATGGCTTTGAACAAGCAGGCGG	Gene cloning
SpLTP1-R	GTGGATCTTGTTGCAGTCGGTGG	
SpLTP1-kb-F	GAGAGGACAGGGTACATGGCTTTGAACAAGCAGGCGG	Vector construction
SpLTP1-kb-R	GTGTCGACTCTAGAGGTGGATCTTGTTGCAGTCGGTG	
q-SpLTP1-F	CTTTGAACAAGCAGGCGGTG	Gene expression analysis
q-SpLTP1-R	CTGTTGAGGTTGCGCACG	
q-GAPDH-F	AGTCCGTCGCCATCGTCA	The reference gene
q-GAPDH-R	CGTGCCCATGCCTTCTGT	

## Data Availability

All data are presented in the article and the Supplementary Materials.

## Supplementary Materials

The following supporting information can be downloaded at https://www.mdpi.com/article/10.3390/plants14203198/s1, Table S1: Nomenclature of LTP Homologous Genes in Different Plants; Table S2: RNA-seq sample sequencing data summary; Table S3: Sequences of qRT-PCR primers; Table S4: Statistics of Differentially Expressed Genes (DEGs) in Transcriptome; Figure S1: Expression analysis of the SpLTP gene family and identification of the *SpLTP1* gene; Figure S2: Volcano plot and PCA analysis of transcriptome comparison between *SpLTP1_OE* and WT; Figure S3: Expression levels of LTP in four *Arabidopsis* lines.
