# Supplementary material for: Overexpression of the Lipid Transfer Protein Gene SpLTP1 from Desert Pioneer Plant Stipagrostis pennata Enhances the Drought Tolerance in Arabidopsis
Source: Plants (Basel). 2025 Oct 18;14(20):3198. doi: 10.3390/plants14203198 (PMC12566629; doi:10.3390/plants14203198)
Supplement: Supplementary file 1 [file plants-14-03198-s001.zip › Figure S1.pdf]

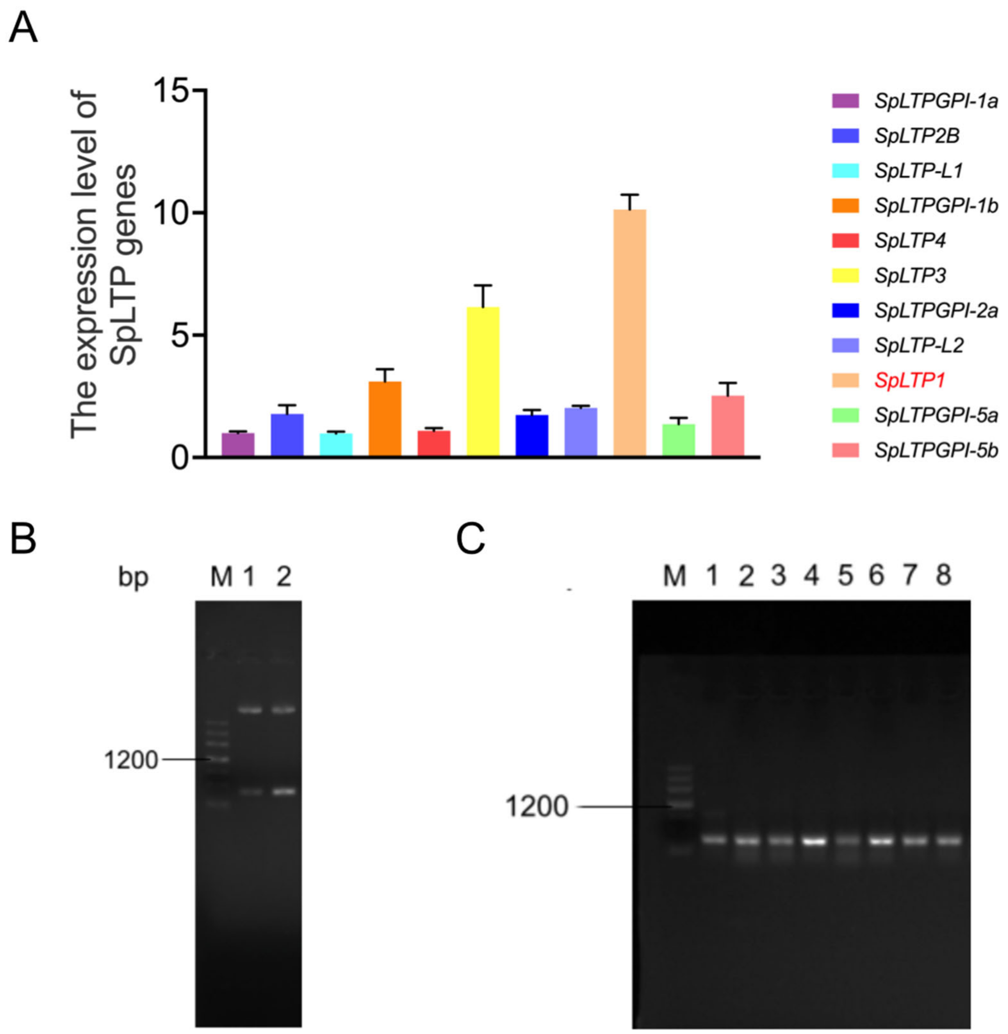

Figure S1. Expression analysis of the SpLTP gene family and identification of the *SpLTP1* gene. (A) Expression of the LTP gene in the rhizosheath roots of *Stipagrostis pennata* at 90 days. (B) Restriction enzyme digestion verification of the *35S::SpLTP1-GFP* vector construct. Lane M: DNA Marker III; Lanes 1-2: *35S::SpLTP1-GFP* construct showing the upper band as *SpLTP1* gene fragment and the lower band as pCAMBIA1300-GFP vector backbone. (B) PCR-based genotyping of *SpLTP1* transgenic Arabidopsis. Lane M: DNA Marker III; Lanes 1-4: *SpLTP1* detection in *SpLTP1-OE* transgenic lines; Lanes 5-8: *SpLTP1* detection in *SpLTP1-atltp1* complementation lines.
