# Supplementary material for: Overexpression of the Lipid Transfer Protein Gene SpLTP1 from Desert Pioneer Plant Stipagrostis pennata Enhances the Drought Tolerance in Arabidopsis
Source: Plants (Basel). 2025 Oct 18;14(20):3198. doi: 10.3390/plants14203198 (PMC12566629; doi:10.3390/plants14203198)
Supplement: Supplementary file 1 [file plants-14-03198-s001.zip › Figure S2.pdf]

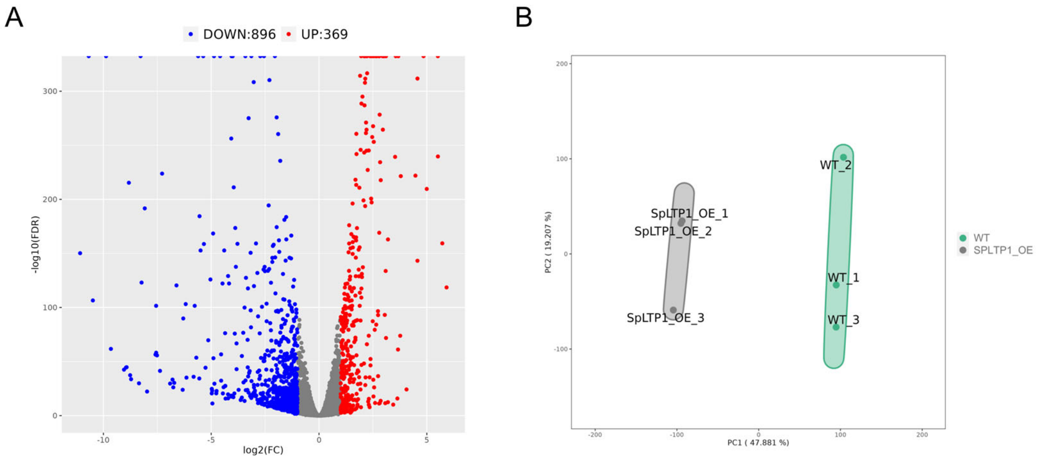

Figure S2. Volcano plot and PCA analysis of transcriptome comparison between *SpLTP1_OE* and WT. (A)Volcano plot of DEGs in *SpLTP1-OE* versus WT plants. Red dots represent significantly upregulated genes, while blue dots denote significantly downregulated genes. (B) PCA plot: The gray dots represent the scatter distribution of *SpLTP1_OE*, and the green dots represent the scatter distribution of WT, with three replicates per group. The farther the distance between two samples, the greater the difference in the distribution of reads between the two samples.
