# Supplementary material for: Overexpression of the Lipid Transfer Protein Gene SpLTP1 from Desert Pioneer Plant Stipagrostis pennata Enhances the Drought Tolerance in Arabidopsis
Source: Plants (Basel). 2025 Oct 18;14(20):3198. doi: 10.3390/plants14203198 (PMC12566629; doi:10.3390/plants14203198)
Supplement: Supplementary file 1 [file plants-14-03198-s001.zip › Figure S3.pdf]

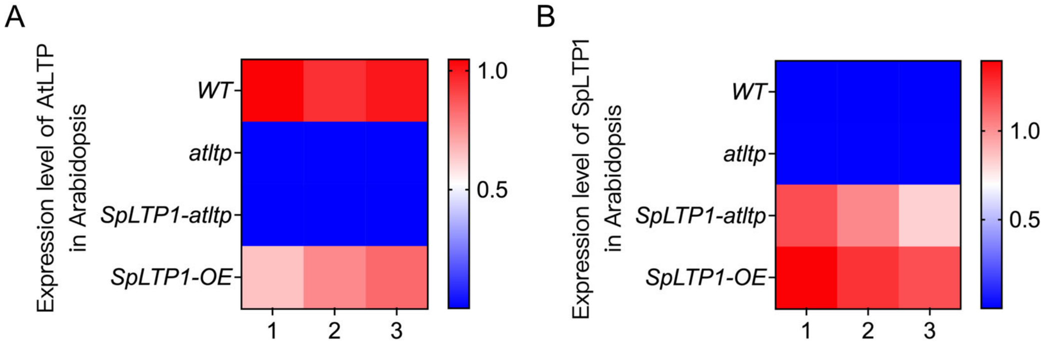

Figure S3: Expression levels of LTP in four *Arabidopsis* lines. (A) Expression level of *AtLTP* in the four *Arabidopsis* lines; The y-axis represents the names of Arabidopsis thaliana lines, the x-axis 1, 2, and 3 represent the expression levels of three independent lines. The right side shows the range of relative expression levels. (B) Expression level of *SpLTP1* in the four *Arabidopsis* lines. The y-axis represents the names of Arabidopsis thaliana lines, the x-axis 1, 2, and 3 represent the expression levels of three independent lines. The right side shows the range of relative expression levels.
