# Supplementary material for: Overexpression of the Lipid Transfer Protein Gene SpLTP1 from Desert Pioneer Plant Stipagrostis pennata Enhances the Drought Tolerance in Arabidopsis
Source: Plants (Basel). 2025 Oct 18;14(20):3198. doi: 10.3390/plants14203198 (PMC12566629; doi:10.3390/plants14203198)
Supplement: Supplementary file 1 [file plants-14-03198-s001.zip › Table S1.pdf]

Table S1 Nomenclature of LTPs Homologous Genes in Different Plant

Species name	code name	GeneID
Phragmites australis	PaLTP1	XP_062201793.1
Arabidopsis thaliana	AtLTP	NC_003076.8
Setaria viridis	SvLTP1	XP_034606507.1
Setaria italica	SiLTP1	XP_004978531.1
Oryza sativa Japonica Group	OsLTP1	XP_015615205.1
Miscanthus floridulus	MfLTP1	XP_066356330.1
Dichanthelium oligosanthes	DoLTP3	OEL13011.1
Musa acuminata	MaLTP1	XP_065003374.1
Phragmites australis	PaLTP3	XP_062181804.1
Lolium rigidum	LrLTP3	XP_047087589.1
Lolium perenne	LpLTP3	XP_051180146.1
Hordeum vulgare subsp. vulgare	HvLTP3	XP_044948717.1
Panicum hallii	PhLTP3	XP_025809252.1
Oryza sativa	OsLTP3	XP_015618578.1
Panicum virgatum	PvLTP3	XP_039775711.1
Hordeum vulgare subsp. vulgare	HvLTP7a2b	KAE8791289.1
Zea mays	ZmLTP3	PWZ30801.1
Zea mays	ZmLTP	ONM42254.1
Oryza sativa	OsLTP	QDX19383.1
Sorghum bicolor	SbLTP3	XP_002442773.2
Setaria italica	SiLTP3	NP_001274465.1
Brachypodium distachyon	BdLTP3	XP_003579021.1
Triticum aestivum	TaLTP3	NP_001414868.1
Triticum urartu	TuLTP3	XP_048535790.1
Triticum dicoccoides	TdLTP3	XP_037433751.1

Oryza brachyantha	ObLTP3	XP_040384543.1
