# Supplementary material for: Overexpression of the Lipid Transfer Protein Gene SpLTP1 from Desert Pioneer Plant Stipagrostis pennata Enhances the Drought Tolerance in Arabidopsis
Source: Plants (Basel). 2025 Oct 18;14(20):3198. doi: 10.3390/plants14203198 (PMC12566629; doi:10.3390/plants14203198)
Supplement: Supplementary file 1 [file plants-14-03198-s001.zip › Table S2.pdf]

Table S2 RNA-seq sequencing data summary

Sample	RawReads	RawBases	CleanReads	CleanBases	Q30	GC
SpLTP1_OE_1	65386538	9807980700	64251058	9481395562	94.09 %	47.03 %
SpLTP1_OE_2	59257126	8888568900	58159986	8543123276	93.91 %	46.92 %
SpLTP1_OE_3	83733454	12560018100	82324410	11971777846	93.96 %	46.59 %
WT_1	46093472	6914020800	45264552	6678533104	94.14 %	46.83 %
WT_2	47638068	7145710200	46855342	6886271558	93.91 %	46.95 %
WT_3	47213948	7082092200	46336940	6790752662	94.08 %	46.66 %

Sample: Sample name; Raw reads: Number of raw sequencing reads; Raw Bases: Total base count of raw sequencing data; Clean reads: Number of filtered reads obtained from raw reads; Clean Bases: Total base count of filtered data; Q30: Percentage of bases with a sequencing error rate < 0.1%; GC content: Percentage of G and C bases relative to total bases.
