# Supplementary material for: Overexpression of the Lipid Transfer Protein Gene SpLTP1 from Desert Pioneer Plant Stipagrostis pennata Enhances the Drought Tolerance in Arabidopsis
Source: Plants (Basel). 2025 Oct 18;14(20):3198. doi: 10.3390/plants14203198 (PMC12566629; doi:10.3390/plants14203198)
Supplement: Supplementary file 1 [file plants-14-03198-s001.zip › Table S3.pdf]

Table S3 Sequences of qRT-PCR primers

Peimer	Primer sequence (from 5' to 3')
AtLTP-F	CAGCCATATCTTGCAACGCC
AtLTP-R	AGACTTGATGCAGCGACACA
AT5G19890-F	GGGGTTAGGGCACAGCTAAG
AT5G19890-R	AAGCAGCCATCCGTATCTCG
AT4G30170-F	GACATGTCATTGGCCGGAGA
AT4G30170-R	CTTTGTTGCGGCAATTGGGA
AT3G01190-F	TGATAGTCAACTACATTTTGATGTTAA
AT3G01190-R	GGCTTTTCAAAATGACTAAACGTTAT
AT4G08780-F	AGGTGGTCACACTTTTGGCA
AT4G08780-R	CAAGGGTTGGGTCGGGTAAA
AT5G17820-F	TCCGAGAAAACTGCTGGACC
AT5G17820-R	TGGCCAATGTGACGATGTCA
AT2G36780-F	GTCCATTGGACCCGTTTCCT
AT2G36780-R	TCTTGATCAATGGCGGCCTT
AT3G23630-F	TTCCCATAGTAGCCGGTGGA
AT3G23630-R	ACGGGTAAGGAAACGTCGAC
AT3G55700-F	TTCTCGTTCGTCGGCTGAAA
AT3G55700-R	ACCGTCTCTCCTCCACCTAC
AT5G19040-F	TCGACGTCTCTAGACCGGTT
AT5G19040-R	GCTCTGGAACTCCAATGGCT
AT1G32640-F	AGAGCCACTAAACCACGTCG
AT1G32640-R	TTGCGTCACCGAGTAACGAA
AT5G57560-F	AACCGCGTGATTTCCAAAGC
AT5G57560-R	AGAAGAAGCCGCGTCTTTGA
AT4G33720-F	TTGACATGTGGGTGGACGAG
AT4G33720-R	TTGCACATCCCAACCTCTCC
AT4G17500-F	CGACAGAGCTGCTTTCAGGA
AT4G17500-R	GGACTTGATTCGAACCGGGT
AT3G12830-F	CGAGATGGAGAGGTTCGTCG
AT3G12830-R	GACAAGGACATGGCAAGGGA
AT5G53590-F	TGCTGTTGATCCTTGGCCTT
AT5G53590-R	ACGATCGACGAGGAGGAAGA
AT4G30290-F	GTCCCGCAAATTCCCAATGG
AT4G30290-R	GCACTCTGGAGGAACACCTC
AT5G48070-F	AAGAACTCCGAAGCCCTTGG
AT5G48070-R	CAATGCTCGGCTTCCCAAAG
SpLTP2B-F	ACGGTGTACAACACGCTGAT
SpLTP2B-R	TTCTTGAGGCAACCGCAGAT
SpLTP-L1-F	CTGCTCTTGGCCATCAACCT
SpLTP-L1-R	TGTTCACCGGGCACTTGTT
SpLTPGPI-1a-F	CCGACTGCCTGTGCTACAT
SpLTPGPI-1a-R	TGACTGATGCAGAGCGAGAC
SpLTPGPI-1b-F	CCACCTGCAACTGCCTCAAG
SpLTPGPI-1b-R	GATGGCGTAAGGGACGGTGA
SpLTP4-F	CTTAGTCGTGATGCTCCTGGT
SpLTP4-R	GAGGCACTTGGGGTTGCG
SpLTP3-F	AGCGGGGTGAAGTCCCTGAAC

SpLTP3-R	CCATGTTGACGCCGGTGAGC
SpLTPGPI-2a-F	GTCAGCGTCGACTACAAGCG
SpLTPGPI-2a-R	CTGCAGATGGCACCAATCCT
SpLTP-L2-F	GTGGACTCGCTGAAGATCGG
SpLTP-L2-R	GAGCTTGATGGTGGTGCAGA
SpLTPGPI-5a-F	GAGATGGAGCGTTGCTCGT
SpLTPGPI-5a-R	TTGAGGCACGAGTACATCCC
SpLTPGPI-5b-F	CTACATCTCCGGGAACGTGT
SpLTPGPI-5b-R	GTGCGGTTGATGTTGAGCC
