# Supplementary material for: Overexpression of the Lipid Transfer Protein Gene SpLTP1 from Desert Pioneer Plant Stipagrostis pennata Enhances the Drought Tolerance in Arabidopsis
Source: Plants (Basel). 2025 Oct 18;14(20):3198. doi: 10.3390/plants14203198 (PMC12566629; doi:10.3390/plants14203198)
Supplement: Supplementary file 1 [file plants-14-03198-s001.zip › Table S4.pdf]

Table S4: Statistics of Differentially Expressed Genes (DEGs) in Transcriptome.

Gene	SpLTP1-O E_1	SpLTP1-O E_2	SpLTP1-O E_3	WT_1	WT_2	WT_3
AT1G62500	0.04	0	0	27.51	30.94	29.09
AT5G33370	0.03	0	0.02	23.62	20.67	24.74
AT4G29030	0.1	0.11	0.62	99.11	100.84	107.58
AT3G01345	0.52	0.47	0.76	30.05	26.38	32.5
AT1G74930	268.69	253.29	242.62	4.29	6.6	6.08
AT3G08770	1.82	2.12	1.95	81.97	86.85	79.03
AT2G42840	4.96	5.62	5.24	160.38	148.25	159.85
AT2G05510	330.1	302.45	324.24	10.23	11.93	11.54
AT5G23940	1.52	1.37	1.57	36.37	34.52	38.69
AT4G02290	1.67	1.46	2.19	31.18	28.86	31.85
AT1G02205	1.68	1.65	1.56	27.9	24.82	26.61
AT4G29780	143.38	144.68	138.9	12.28	10.57	11.09
AT2G30020	123.53	123.86	122.24	11.2	9.65	11.04
AT1G52400	12.1	11.04	11.99	128.33	121.38	131.78
AT1G70830	15.23	13.93	14.63	134.41	129.56	127.28
AT1G18300	283.21	278.87	292.93	33.35	31.98	35.49
AT4G25470	89.83	86.14	89.77	10.88	12.2	10.17
AT2G40000	611.72	622.95	609.35	76.96	80.02	77.12
AT4G37610	71.53	74.01	81.35	10.6	9.5	9.81
AT1G73540	239.93	252.87	255.73	37.3	38.27	33.59
AT5G66650	74.45	74.41	77.83	11.25	11.17	11.04
AT1G28290	14.91	13.5	13.69	97.33	92.76	91.86
AT1G76650	132.6	140.71	148.62	20.08	23.19	23.46
AT4G24570	299.38	294.75	288.29	48.36	48.26	48.14
AT4G03210	20.85	20.29	20.23	120.42	123.34	121.33
AT3G19580	76.28	77.78	79.98	13.16	14.61	13.27
AT1G74450	93.64	91.58	101.78	18.28	17.34	17.47
AT1G60190	54.35	53.02	52.72	10.83	10.45	10.17
AT1G27730	443.13	435.98	428.68	94.75	95.37	94.01
AT3G44260	298.11	305.52	319.95	69	70.06	64.01
AT3G46620	242.31	239.75	235.44	54.96	54.48	57.73
AT3G55980	152.43	153.82	150.8	37.43	35.67	34.15
AT1G23710	115.26	120.55	121.61	28.41	28.31	28.53
AT3G01420	12.3	11.55	12.11	51.19	48.48	51.23
AT1G18740	185.27	184.57	191.08	47.54	47.7	51.73
AT1G80840	68.87	66.75	70.53	15.21	14.75	14.62
AT2G20670	310.6	299.39	328.5	83.1	81.57	89.72
AT4G34410	38.23	35.36	35.57	1.8	1.45	1.42
AT5G59820	273.74	273.11	261.44	62.3	61.35	61.85
AT5G54510	7.63	7.97	7.73	38.8	36.03	41.67
AT2G33850	6.5	5.58	6.88	54.91	52.13	51.3

AT2G27080	153.36	147.49	159.99	36.12	32.46	38.32
AT4G32480	133.77	137.93	134.53	33.41	32.56	36.16
AT4G11280	105.5	104.88	114.89	27.32	26.88	30.28
AT2G24550	153.93	155.45	166.17	40.49	33.81	37.56
AT5G45630	118.83	126.85	123.23	18.56	16.66	18.09
AT4G21650	13.6	12.72	13.77	54.71	49.33	55.18
AT5G45670	2.35	2.22	2.82	25.12	23.51	24.45
AT2G30040	42.26	41.85	41.07	9.37	9.3	9.34
AT3G04640	166.56	166.21	196.42	29.41	32.71	32.35
AT2G35930	51.71	47.2	45.4	6.48	5.94	6.4
AT5G22250	134.69	141	141.07	29.96	33.44	28.34
AT5G61600	140.88	132.87	132.26	32.01	30.44	28.74
AT1G13260	269.6	271.27	267.02	85.87	80.36	80.95
AT4G29020	48.05	50.58	48.01	189.97	178.68	183.73
AT1G32928	181.27	194.78	194.61	32.91	35.37	36.42
AT5G24770	1.08	1.43	1.39	22.86	21.56	22.39
AT1G28370	79.1	75.03	78.97	11.99	14.28	14.41
AT5G59550	107.63	104.37	99.98	28.46	27.14	27.54
AT1G07135	148.34	149.65	166.44	28.92	35.05	31.24
AT5G04340	172.16	168.61	160.29	34.71	39.49	36.07
AT3G61190	119.18	127.32	130.55	31.92	30.02	27.64
AT1G22190	211.01	211.59	204.39	64.06	62.54	64.17
AT5G21960	24.68	24.51	24.34	0.6	0.57	0.44
AT1G33760	53.78	49	53.43	4.81	5.56	3.35
AT3G04290	20.85	20.14	20.75	73.32	68.5	76.22
AT4G27652	282.03	302.7	260.62	39.4	41.46	39.36
AT1G21910	74.99	77.76	86.02	17.15	14.97	18.63
AT1G11600	0.09	0.05	0.02	7.88	8.63	8.42
AT4G27654	81.98	88.74	90.84	2.38	5.04	4.44
AT1G15010	98.22	89.94	81.78	5.43	7.41	6.88
AT1G35140	138.65	137.67	136.07	44.23	43.21	42.25
AT3G52450	22.84	24.57	23.67	3.19	2.93	3.79
AT2G20870	0.08	0.09	0	26.95	29.27	30.06
AT2G28400	146.21	145.11	149.64	43.75	45.87	45.68
AT2G28790	1.43	2	1.74	24.46	28.6	28.12
AT1G32640	128.95	124.04	121.51	34.21	36.52	33.13
AT1G66270	11.74	10.62	12.99	0.43	0.36	0.33
AT4G27657	317.85	330.38	303.19	51.59	62.3	66.78
AT3G16510	40.89	40.34	43.12	10.84	9.23	10.2
AT5G39120	41.95	42.27	43.79	7.36	8.07	8.51
AT5G57560	324.82	326.58	343.23	107.91	112.04	123.56
AT3G53190	4.65	4.17	4.43	23.29	22.75	21.88
AT3G02840	40.67	38.93	39.6	8.6	9.76	9.07

AT5G22430	0	0	0.16	20.75	18.37	20.37
AT1G52030	0.14	0.15	0.05	5.24	5.49	5.16
AT4G18970	18.21	18.49	20.03	55.43	54	56.62
AT1G30700	13.31	13.15	13.91	41.53	42.27	41.04
AT2G40140	91.08	94.31	94.33	35.61	34.25	37.97
AT5G51750	4.48	4.52	4.9	19.48	17.45	18.28
AT3G23290	1.53	2.15	1.34	25.74	24.64	23.25
AT2G22500	336.43	332	312.68	106.03	94.84	93.54
AT4G13395	234.54	259.13	276.49	32.34	46.45	33.74
AT1G70260	0.34	0.31	0.19	8.17	8.08	8.87
AT3G56880	250.91	237.67	240.25	91.96	83.7	86.35
AT1G50010	59.14	56.89	60.5	148.54	140.39	148.31
AT1G72920	70.5	72.91	72.09	24.26	24.61	23.18
AT2G36220	201.93	197.35	187.6	63.15	61.12	56.59
AT5G11070	220.49	212.33	207.24	68.8	70.55	66.83
AT1G64390	15.24	13.94	14.68	44.03	40.91	42.57
AT5G45340	18.84	19.99	24.49	2.47	2.21	2.32
AT2G36570	5.49	5.91	5.7	21.26	21.15	20.41
AT5G21940	195.46	204.37	183.78	60.53	61.24	57.81
AT1G19770	179.18	173.25	188.55	66.4	55.58	64.43
AT5G65080	14.77	14.13	16.05	0.14	0.35	0.35
AT4G22490	7.06	6.23	7.03	53.32	54.32	49.47
AT3G15450	195.17	202.37	194.83	78.1	75.46	75
AT5G20740	1.44	0.96	1.58	19.27	19.92	17.86
AT4G30250	0.22	0.12	0.12	7.06	5.94	6.2
AT3G29030	9.15	8.69	9.47	39.16	41.25	39.01
AT4G28250	8.65	7.76	7.73	34.31	36.81	36.68
AT5G51190	104.24	107.92	100.67	26.51	31.57	27.96
AT2G41010	93.39	88.1	97.93	30.92	31.28	33.68
AT1G73500	178.21	175	169.39	66.71	63.61	62.69
AT3G46090	196.55	197.98	180.67	50.52	57.79	55.05
AT5G04950	0.72	0.44	0.59	11.79	11.84	14.34
AT5G08000	0.3	0.28	0.27	14.19	13.2	12.22
AT4G38860	29.51	32.08	33.02	110.6	114.76	119.82
AT5G64260	331.31	319.04	310.87	127.88	124.45	125.3
AT1G75240	2.35	1.73	1.55	18	15.78	17.21
AT1G62360	0	0	0	10.64	8.58	8.37
AT1G80440	142.04	144.45	137.06	50.7	49.4	45.75
AT5G07030	8.4	7.01	8.27	34.36	33.47	30.99
AT5G47230	40.61	42.05	44.68	13.75	12.57	13.29
AT5G15780	37.04	37.43	36.99	100.56	90.82	100.67
AT1G70710	5.29	5.47	6.29	28.4	24.93	24.7
AT1G72970	19.84	20	20.88	50.68	48.8	52.09

AT1G26390	23.63	24.44	28.97	76.86	73.92	78.55
AT4G25490	23.08	25.69	21.09	1.36	0.51	1.11
AT1G67750	4.91	4.63	5.22	23.72	20.37	23.48
AT1G12570	0.5	0.36	0.39	6.07	5.68	6.79
AT1G18250	4.29	3.81	3.77	23.23	23.27	22.49
AT1G66400	68.08	77.11	77.62	19.65	17.84	17.79
AT4G34760	10.79	11.84	12.73	62.28	58.55	58.21
AT2G38390	2.61	2.19	2.74	14.65	15.13	16.15
AT5G60680	223.86	226.23	197.65	61.33	64.01	58.18
AT1G24020	37.29	39.42	30.78	180.46	205.98	174.85
AT1G72910	97.81	96.24	105.35	40.53	38.63	36.75
AT5G17350	106.57	104.09	98.6	10.02	16.59	10.1
AT1G10640	1.88	1.75	2.1	10.92	11.6	10.9
AT2G42610	7.13	6.61	6.96	47.97	57.67	46.74
AT1G02660	16.96	15.11	15.95	3.94	3.97	4.4
AT5G55730	9.61	8.54	10.02	30.51	29.06	31.9
AT3G49110	0.16	0.54	0.56	7.7	8.29	8.5
AT3G62550	98.7	99.46	111.03	30.46	35.07	30.78
AT1G25400	44.17	47.25	46	11.13	13.37	14.01
AT1G72940	48.46	47.29	48.36	17.39	18.72	17.63
AT3G07350	33.39	32.54	31.97	8.65	8.65	7.65
AT5G24780	1.37	1.2	1.82	14.5	17.11	16.18
AT4G08150	0.17	0.26	0.18	6.44	6.25	7.68
AT2G25250	156.4	145.88	159.89	57.74	60.31	56.18
AT5G47070	38.36	37.52	39.39	14.52	13.79	15.53
AT5G20630	91.3	91.06	95.39	206.32	209.02	212.54
AT3G05727	0	0	0.22	28.75	36.54	34.3
AT1G78490	2.46	2.59	2.69	12.99	11.78	12.14
AT3G05600	0.24	0.58	0.41	7.42	7.24	7.67
AT3G52910	0.42	0.23	0.21	6.24	6.42	6.96
AT1G76600	84.3	85.9	85.44	34.8	34.6	34.75
AT2G17040	97.71	99.6	104.38	42.91	43.3	44.53
AT1G57750	0.02	0.1	0.02	4.22	4.54	5.2
AT5G47500	1.4	1.09	1.05	9.35	9.61	9.93
AT2G34810	21.87	22.52	22.8	51.62	50.53	54.76
AT4G27280	388.36	397.31	373.43	143.68	153.26	138.16
AT1G19210	12.41	11.89	12.69	0.43	0	0.17
AT5G58430	71.13	71.45	69.85	30.89	30.13	32.97
AT1G66160	32.17	35.69	37.16	11.87	10.56	12.35
AT1G06080	1.01	1.08	1.48	12.94	12.41	11.11
AT3G50060	71.08	67.82	73.52	24.63	25.94	29.44
AT3G29000	44.51	48.52	50.18	12.77	13.34	11.07
AT3G28340	44.46	42.86	49.77	15.77	16.43	16.35

AT5G56550	58.71	58.23	66.24	18.41	14.5	17.25
AT1G06360	7.2	7.08	9.67	34.54	31.1	34.16
AT3G52400	197.96	195.93	193.36	90.4	88.58	85.28
AT1G68840	264.14	259.18	243.06	103.98	106.65	101.83
AT3G19030	382.76	444.96	416.77	116.11	139.42	136.12
AT5G23020	27.09	26.1	27.35	10.72	10.32	10.62
AT4G33720	44.76	49.04	46.65	13.01	11.97	11.52
AT1G57990	53.69	54.87	57.69	24.28	24.34	25.34
AT5G67070	33.12	32.36	31.65	102.87	109.75	97.82
AT1G32920	386.42	425.79	403.43	159.24	170.54	166.8
AT2G43800	4.37	3.77	4.05	12.95	12.36	12.35
AT4G01950	48.98	48.42	48.43	22.12	19.43	20.45
AT4G01250	39.6	39.89	40.23	15.36	12.61	14.6
AT5G37940	0	0	0	6.35	5.68	7.8
AT1G19380	86.33	90.19	89.71	35.4	33.57	32.18
AT1G62480	122.81	119.75	114.85	308.29	332.59	302.69
AT4G37750	1.83	1.36	1.68	8.46	8.54	9.79
AT4G22517	0.2	0.11	0.23	14.36	14.77	15.33
AT2G29550	28.88	27.96	28.98	64.79	60.78	63.54
AT2G21140	17.74	16.8	17.86	48.26	42.95	45.61
AT4G37260	71.23	70.14	64.58	23.22	25.67	23.1
AT1G58420	48.67	52.99	51.1	12.85	16.01	12.97
AT5G44620	0.02	0	0.02	3.27	3.18	3.3
AT1G06350	0.08	0.13	0.09	5.43	5.59	5.8
AT4G25620	44.7	43.67	43.55	19.3	18.66	20.15
AT5G19120	80.07	78.06	73.92	29.25	29.78	26.09
AT4G38840	8.08	8.57	9.5	49	54.67	63.35
AT2G45570	13.39	12.26	12.83	32.21	29.75	32.31
AT5G28640	1.37	0.91	0.99	12.19	11.22	12.94
AT3G11650	53.3	51.62	54.98	20.73	21.72	20.16
AT5G64660	28.35	28.18	26.92	9.72	10.03	10.18
AT5G24590	47.85	48.17	49.14	20.14	19.75	23.08
AT2G18700	35.49	37.69	37.61	16.73	17.34	16.91
AT4G24380	34.66	34.12	33.82	12.01	11.98	12.48
AT5G62700	51.85	54.26	53.34	116.31	107.34	112.3
AT1G73260	84.96	91.15	82.07	205.34	216.55	208.38
AT3G18000	4.03	4.78	4.61	15.25	14.34	16.2
AT4G30140	0.76	0.81	0.93	8.02	7.77	7.37
AT1G31710	2.81	3.11	2.82	11.45	10	12.11
AT3G10930	175.27	184.63	131.23	25.21	25.96	23.81
AT2G21050	2.36	2.91	3.05	11.53	11.07	11.15
AT3G49670	10.4	10.92	10.51	26.96	24.15	24.76
AT3G12145	5.42	6.75	5.94	23.96	21.26	22.35

AT1G09070	944.48	914.51	918.15	438.13	448.87	418.11
AT1G15520	21.61	21.27	22.3	47.57	42.3	46.9
AT3G16720	74.92	75.16	70.22	30.8	30.59	32.01
AT5G62020	49.01	50.07	49.62	22.31	20.72	21.27
AT3G48360	7.96	8.78	8.33	0.8	0.89	1.36
AT4G17500	149.57	146.43	140.7	59.33	56.28	49.58
AT5G51390	29.87	33.31	31.65	5.53	4.65	4.18
AT3G55970	7.97	8.14	8.27	23.44	21.64	22.97
AT3G62720	43.31	41.49	42.18	19.75	17.94	18.32
AT1G01600	0.85	0.65	0.81	5.77	5.75	5.29
AT2G41640	31.76	29.52	31.42	13.45	13.31	13.17
AT3G50930	81.71	83.51	85.33	42.12	39.55	44.66
AT2G45220	54.89	51.66	51.23	114.3	108.33	111.94
AT3G49530	44.6	47.26	48.04	21.89	20.14	22.69
AT3G57450	397.29	418.74	412.76	173.04	191.57	172.35
AT5G63090	0.12	0	0.15	7.78	8.41	9
AT2G29470	9.91	8.83	9.8	30.55	29.08	32.88
AT2G27500	50.86	51.74	54.87	25.53	22.63	25.03
AT2G45470	48.22	49.23	49.32	119.14	110.47	105.44
AT3G50800	43.15	43.03	45.63	11.31	13.34	14.63
AT3G47340	72.11	70.02	70.81	36.94	34.7	35.68
AT5G37770	207.6	208.66	227.11	78.16	91.92	94.28
AT3G23250	17.15	17.34	18.28	4.81	4.47	4.83
AT3G23730	9.33	8.58	9.91	26.86	26.51	29.36
AT1G09080	13.4	13.58	15.6	4.9	4.12	4.46
AT1G78100	22.29	22.82	21.52	51.26	48.77	50.24
AT1G61470	13.86	14.57	16.56	2.84	2.46	2.85
AT5G62210	3.56	3.31	3.47	19.13	20.03	17.36
AT1G27100	124.05	119.02	117.42	55.26	57.18	52.67
AT1G35210	56.97	53.58	58.41	17.51	20.73	17.9
AT2G24600	23.45	24.89	25.92	9.99	10.94	10.23
AT2G27830	96.68	95.65	89.59	38.27	37.43	41.89
AT4G02850	3.2	2.37	2.89	12.85	14.05	14.53
AT1G70090	75	72.62	75.52	34.9	36.53	37.36
AT4G38660	9.58	8.27	8.44	28.51	26.08	25.95
AT5G22630	55.57	57.05	53.48	26.23	23.91	24.93
AT1G66280	8.07	9.16	10.3	1.54	1.13	1.78
AT4G17490	42.45	40.34	38.96	13.66	15.41	13.63
AT2G36780	15	16.18	16.49	5.43	5.23	5.97
AT5G63130	41.44	41.53	41.05	14.33	11.78	15.3
AT5G16250	8.47	8.75	9.98	30.58	31.74	30.8
AT1G10340	35.98	32.95	34.24	15.39	13.24	15.52
AT1G67340	39.18	40.81	40.29	18.34	18.49	18.97

AT2G26330	7.42	8.45	8.82	19.31	18.22	20.52
AT3G17840	10.58	9.71	9.53	24.23	23.82	23.4
AT3G56360	593.69	567.02	537.96	257.2	263.39	246.7
AT1G52040	0.65	0.47	0.78	6.18	5.08	6.41
AT1G13650	5.55	7.03	6.92	22.66	21.94	21.59
AT3G46600	53.06	54.23	58.32	26.91	22	25.07
AT3G56370	11.18	10.84	11.12	26.44	24.45	30.29
AT4G23820	21.97	21.81	22.68	46.73	45.01	44.92
AT1G72950	7.74	7.84	7.71	1.19	1.23	1.5
AT5G08240	40.95	42.9	45.67	18.73	15.64	16.73
AT3G45860	67.32	66.25	74.17	35.21	31.47	35.65
AT1G14430	1.14	1.02	1.47	5.98	5.71	6.01
AT5G48360	5.48	5.11	5.79	14.33	13.33	13.3
AT3G52800	458.55	464.6	424.33	212.95	212.6	203.24
AT1G68140	44.06	43.58	42.33	88.62	88.48	87.91
AT4G23800	2.73	2.66	2.85	9.38	9.61	10.11
AT5G37300	0.4	0.29	0.26	3.54	3.83	3.63
AT2G42800	0.12	0.3	0.25	5.66	4.51	4.18
AT1G68238	5.07	7.36	7.54	41.65	49.09	46.67
AT4G33790	0.16	0.23	0.26	3.6	3.09	3.5
AT1G19670	21.17	20.14	21.23	45.13	47.45	45.68
AT2G44840	20.03	20.61	17.93	3.99	4.32	3.64
AT1G12610	9.12	10.23	9.39	0.54	1.04	0.53
AT2G37640	8.76	8.25	7.26	27.14	25.5	24.58
AT5G65300	36.69	37.93	36.48	10.82	12.38	11.67
AT5G65070	6.94	6.68	6.86	1.02	0.54	0.85
AT3G22620	9.11	8.15	10.2	30.36	26.95	30.2
AT5G22880	65.93	63.05	65.79	152.95	150.32	136.8
AT2G44240	6.07	7.8	8.06	22.03	20.56	21.71
AT4G12880	38.74	38.18	39.79	82.01	86.43	90.56
AT1G68220	12.17	10.7	8.95	35.7	34.54	36.94
AT5G58390	0.8	1.05	0.97	8.77	11.2	8.46
AT5G22380	21.22	21.34	23.22	6.37	7.13	7.27
AT4G32810	4.31	4.07	4.41	11.4	12.42	11.99
AT1G63710	0.04	0.05	0.12	2.68	3.21	2.55
AT2G33580	29.28	27.72	32.22	14.52	13.33	14.49
AT1G80080	2.14	2.57	2.2	11.91	11	9.39
AT2G44940	9.12	8.47	8.92	23.71	22.63	25.85
AT1G64065	47.99	44.9	47.16	18.35	16.16	20.97
AT2G23170	1.72	1.61	1.38	6.48	6.25	6.1
AT2G16660	5.26	5.76	6.04	16.46	14	15.95
AT1G80280	2.39	2.01	2.18	7.66	6.95	8.18
AT4G08040	12.02	12.73	12.1	4.1	3.55	4.37

AT3G62260	42.06	41.01	42.57	21.8	20.47	19.71
AT3G23890	0.75	0.6	0.78	2.74	2.49	2.69
AT3G02640	4.47	4.88	5.25	18.87	20.77	19.46
AT3G44300	45.38	44.97	41.1	90.17	96.8	97.92
AT2G41850	0.19	0.21	0.35	4.14	3.27	3.91
AT4G28040	25.68	25.96	28.56	11.27	11.85	11.25
AT2G22880	38.34	46.03	39.72	9.14	10.29	7.46
AT2G14890	51.39	54.42	48.08	115.06	115.53	109.3
AT2G04780	23.22	22.26	26.07	55.36	53.93	51.8
AT3G49340	3.1	2.73	3.04	11.25	10.02	11.09
AT5G62470	26.45	26.7	29.97	12.14	11.12	12.46
AT2G46510	22.37	23.27	24.52	11.21	11.62	11.56
AT4G28780	25.4	23.31	24.08	51.28	48.5	48.66
AT3G15210	254.95	258.4	207.24	86.51	90.66	84.51
AT2G26530	44.86	44.14	44.22	21.41	22.53	20.97
AT3G20600	51.46	53.52	51.18	24.26	24.5	23.09
AT4G05070	189.91	191.63	173.48	75.15	82.13	69.64
AT1G20823	62.89	59.41	55.36	25.07	23.68	25.18
AT4G28680	0	0	0	2.53	1.7	1.98
AT2G46400	50.18	56.37	58.91	25.84	26.21	26.39
AT3G28550	1.54	1.48	1.65	0.16	0.06	0.15
AT1G19250	8.19	7.92	9.18	20.19	18.25	21.68
AT3G13510	15.94	16.65	14.17	34.77	37.01	36.93
AT3G13980	2.48	2.46	2.07	9.3	9.48	11.31
AT2G42200	1.47	2.38	1.9	9.42	8.2	8.7
AT2G21210	2.16	2.93	2.89	13.35	14.73	14.73
AT3G10020	134.93	124.21	124.59	55.37	55.13	44.84
AT2G23000	2.27	2.66	2.31	8.53	8.25	8.05
AT2G25060	4.41	5.8	4.79	20.76	26.47	23.25
AT1G07790	32.22	31.58	31.32	72.82	83.42	75.23
AT5G62080	0	0.13	0	10.61	9.73	10.75
AT1G25560	198.47	194.81	176.03	88.53	95.56	90.04
AT5G22580	78.11	80.36	69.61	196.06	244.9	207.39
AT2G03090	5.82	7.15	5.91	18.91	18.15	20.44
AT2G34500	8.82	8.84	9.79	20.86	20.07	19.4
AT5G52860	0.08	0.04	0.11	2.19	1.8	1.86
AT3G13960	0	0	0.02	2.88	2.39	2.39
AT3G49790	18.74	18.36	19.76	8.5	6.49	7.57
AT3G54200	40.74	37.6	40.62	19.18	17.69	18.5
AT1G75780	0.88	1.03	0.85	5.23	4.59	5.85
AT5G47850	6.81	6.87	6.29	2.15	2.2	1.71
AT3G12110	9.68	9.61	9.38	21.6	24.6	23.81
AT5G37950	0	0	0.03	2.57	2.7	2.98

AT5G23530	1.37	1.71	1.13	6.88	7.14	6.95
AT2G32990	3.6	4.16	4.14	13.16	10.66	11.77
AT1G66500	15.03	15.27	15.33	6.51	5.3	6.55
AT1G53830	0.18	0.22	0.26	2.48	2.44	3.24
AT3G44990	7.3	7.65	8.37	20.24	19.44	19.33
AT2G15890	24.95	24.88	27.71	9.19	10	9.73
AT2G01610	3.78	3.55	3.45	12.98	12.36	13.1
AT1G02730	2.42	2.5	2.41	7.58	6.26	6.35
AT2G04570	10.42	11.11	11.23	25.98	22.68	26.51
AT5G14230	1.18	1.17	1.29	4.13	3.89	4.22
AT2G42760	19.21	18.08	18.09	6.88	6.39	5.57
AT3G55110	2.95	3.12	2.56	8.14	7.84	7.79
AT1G02800	0	0.05	0.05	2.32	1.61	2.25
AT4G09160	3.92	3.96	3.45	10.01	9.29	9.52
AT1G72230	2.79	3.44	2.39	15.73	19	15.09
AT3G15680	12.45	11.86	10.41	31.07	32.28	32.91
AT5G22500	0.7	0.7	0.73	5.46	4.37	4.2
AT3G21500	2.54	2.7	2.83	8.37	7.24	7.4
AT5G22740	11.11	10.42	10.24	23.39	20.7	23.24
AT1G04660	0.05	0.12	0.25	4.18	5.27	5.07
AT1G15570	1.05	1.16	1.48	5.84	5.28	5.35
AT3G60140	1.03	1.39	1.16	4.88	4.28	4.67
AT2G01300	14.48	14.58	13.48	2.99	2.58	2.63
AT4G35770	23.23	26.28	25.36	8.83	9.11	9.66
AT3G10570	2.22	2.24	2.53	7.77	7.06	7.01
AT4G34160	6	5.49	5.23	15.57	15.86	14.53
AT3G12830	76.8	74.6	68.07	28.94	32.09	31.63
AT4G36180	1.29	1.42	1.22	4.14	3.58	3.94
AT4G21680	8.74	10.81	9.8	23.73	20.49	22.79
AT5G55180	9.04	8.12	8.62	20.51	19.12	18.67
AT1G58225	48.39	52.71	54.84	23.59	24.1	24.67
AT5G28490	1.87	2.61	1.42	11.94	11.17	10.64
AT3G57010	2.6	3.26	3.43	9.44	10.33	10.32
AT1G59590	46	50.18	54.56	23.42	23.8	23.81
AT1G65450	0.57	0.5	0.7	3.73	3.69	3.49
AT3G25780	33.82	33.32	35.34	17.86	15.03	15.69
AT3G59080	19.75	18.92	21.65	8.05	8.22	10.22
AT3G21190	7.97	7.71	7.53	16.67	17.56	18.54
AT3G25600	67.32	73.08	69.56	30.74	35.67	31.95
AT4G29360	1.31	1.48	1.39	5.19	5	4.81
AT3G61060	25.73	27.5	28.03	13.57	12.38	12.72
AT3G46080	75.89	86.78	69.01	28.13	30.49	25.79
AT4G23160	2.89	3.02	3.24	1.1	1.01	1.14

AT1G10560	4	3.59	4.17	0.72	0.91	1.18
AT3G09520	11.8	12.26	11.8	5.81	5.55	5.55
AT1G17020	6.2	6.87	6.68	15.94	14.92	17.34
AT1G76540	2.76	3.05	3.02	9.73	8.73	10.01
AT2G35290	34.81	31.54	28.76	8.48	9.19	6.9
AT4G15920	8.58	7.24	7.06	20.51	22.72	25.06
AT5G54490	47.25	54.54	56.17	16.3	19.71	22.08
AT1G15100	18.94	17.13	17.68	42.08	47.03	43.05
AT1G12010	12.22	11.35	12.14	3.98	4.42	3.31
AT3G18850	1.65	1.53	1.61	7.05	5.68	6.68
AT1G23720	2.06	2	2.09	0.33	0.32	0.53
AT1G54740	25.74	25.19	25.81	12.56	12.82	12.6
AT1G61340	29.78	34.16	36.42	13.74	12.9	13.23
AT5G65410	10.18	11.19	9.92	25.29	26.7	23.98
AT4G01360	21.23	21.39	23.76	8.9	8.84	11.24
AT5G16200	45.74	42.41	44.56	14.59	20.04	16.84
AT2G16850	3.6	3.67	4.18	11.01	11.56	12.86
AT5G52160	0	0	0	8.67	7.39	8.35
AT1G12080	14.95	12.97	13.4	34.55	35.41	34.27
AT3G50890	0.32	0.72	0.4	4.4	4.67	5.08
AT4G20940	1.58	1.64	2.04	5.2	4.41	5.19
AT1G31310	0	0	0	2.27	1.78	2.23
AT5G51950	0.02	0.02	0.05	1.38	1.33	1.22
AT5G41750	14.46	14.18	15.67	7.56	5.46	7.14
AT1G67470	25.45	26.72	27.41	13.6	11.3	14.45
AT5G23820	27.21	31.3	30.8	62.82	68.25	61.49
AT5G53590	68.28	74.12	79.88	34.15	29.41	38.53
AT4G26760	1.77	1.94	1.72	5.5	5.25	5.24
AT5G15210	13.11	13.49	14.33	28.42	26.17	29.18
AT1G64360	82.4	75.67	80.77	36.85	38.4	36.18
AT1G69690	10.3	8.75	9.79	22.12	19.97	21.77
AT1G14900	9.4	8.67	8.65	23.11	23.33	21.68
AT5G59970	35.29	35.53	33.83	82.56	100.79	84.42
AT4G33270	1.16	1.03	1.22	4.31	4.42	4.71
AT5G10400	25.69	29.77	26.8	60.79	72.08	65.59
AT1G24070	0.04	0.11	0.17	1.49	1.72	1.68
AT3G22790	0.14	0.22	0.24	1.04	1.02	1.29
AT2G41820	2.07	2.47	2.75	6.61	5.84	7.11
AT1G27710	0	0	0	4.1	4.75	3.21
AT4G03510	17.87	18.62	19.61	6.21	8.1	7.49
AT1G28390	2.35	2.71	2.61	7.93	6.91	7.05
AT1G25510	0.72	0.74	0.78	3.34	3.57	3.58
AT1G57820	4.32	4.61	4.31	9.56	9.96	9.56

AT2G38110	4.53	3.79	4.54	10.37	9.65	10.44
AT5G57510	21.06	22.54	20.92	5.31	5.71	7.76
AT1G09500	0.56	0.54	0.52	3.89	3.51	3.82
AT2G32030	26.62	27.94	25.74	10.87	11.46	11.17
AT1G02065	0.03	0	0	3.31	2.2	2.1
AT5G46710	40.91	42.98	47.06	19.79	19.31	24.2
AT4G31805	0.54	0.26	0.26	3.23	3.02	3.68
AT1G08560	2.78	3.29	3.05	9.51	9.01	8.88
AT1G77640	12.25	11.48	14.62	3.5	3.43	4.08
AT3G28500	2.19	1.32	1.79	10.87	14.1	14.1
AT2G40970	31.98	31.83	28.29	13.05	13.89	11.92
AT3G10985	172.71	177.61	152.53	80.12	83.91	72.37
AT5G63560	1.11	1.35	1.1	4.58	4.34	4.8
AT5G43020	3.1	3.05	2.94	8.11	7.02	7.18
AT4G32460	8.58	7.06	7.81	20.19	18.64	17.48
AT1G65790	7.98	7.82	8.69	3.98	3.98	4.26
AT3G27650	0.94	0.4	0.79	6.16	7.69	7.24
AT1G18650	10.03	10.25	9.59	23.6	24.6	22.71
AT1G23205	5.04	5.4	4.08	14.52	14.9	14.72
AT5G11550	5.5	6.25	5.46	17.25	19.38	15.17
AT1G07610	21.58	23.9	23.4	63.63	66.7	63.85
AT4G14450	18.04	18.77	20.31	6.29	5.07	5.92
AT4G37390	2.35	2.58	2.53	6.53	6.06	6.1
AT5G64870	11.52	11.05	10.87	4.14	5.19	4.53
AT5G22940	2.38	1.95	2.3	6.25	6.02	6.11
AT4G02060	3.81	4.08	4.09	8.59	7.84	8.84
AT1G21880	5.99	5.62	6.44	16.31	12.88	16.47
AT3G15760	35.3	37.98	39.3	17.72	16.84	16.23
AT2G30210	1.6	1.7	1.79	5.55	5.07	4.78
AT2G38820	22.52	19.79	22.24	9.65	10.09	11.03
AT4G31840	8.43	9.55	6.86	24.07	25.9	23.73
AT1G68190	11.33	13.32	13.66	28.32	25.7	33.19
AT5G65207	55.21	67.9	71.13	20.76	22.31	25.32
AT1G49130	10.35	10.03	11.71	3.86	4.28	3.83
AT4G39860	11.66	11.76	11.88	25.66	24.87	22.92
AT1G69120	0	0	0	2.1	2.78	2.95
AT1G49320	2.59	2.18	2.7	8.46	10.31	8.81
AT4G23880	29.43	28.77	30.07	14.31	15.03	13.26
AT5G51560	3.2	2.87	3.27	6.77	6.79	7.3
AT1G23380	0.45	0.66	0.47	3.33	3.63	3.56
AT4G03100	1.18	1.36	1.07	4.5	4.23	4.49
AT1G68185	6.48	5.51	5.59	14.32	14.45	16.36
AT1G09950	5.25	5.26	5.44	1.12	0.54	0.76

AT5G66230	1.26	1.21	1.11	3.92	3.72	4.4
AT1G78970	0.14	0.19	0.14	1.73	1.17	1.72
AT3G16860	10.64	10.32	10.45	5.49	4.31	5.41
AT5G03150	0.94	0.89	1.04	4.52	3.37	4.41
AT5G26730	0	0	0.1	3.31	2.49	2.12
AT3G54560	25.86	23.7	22.71	48.51	54.23	53.89
AT4G37740	1.21	1.55	1.43	4.74	4.24	4.32
AT4G14770	1.24	1.06	0.87	3.98	3.49	3.54
AT4G23700	4.63	5.23	5.29	10.38	9.6	12.03
AT5G58680	5.8	6.07	6.95	1.9	1.91	1.46
AT2G05440	337.68	334.39	394.61	141.57	186.1	150.37
AT3G20260	1.21	1.14	1.09	4.02	4.55	5.15
AT3G23630	4.44	4.42	4.25	0.74	0.94	0.91
AT1G58340	1.93	1.89	2.1	5.73	5.23	5.21
AT5G15530	3.07	3.6	2.93	10.3	9	10.05
AT2G34600	10.77	10.98	12.81	2.6	2.2	3.09
AT2G25735	49.05	51.89	53.21	25.33	22.71	28.18
AT1G58430	0	0.04	0.03	2.06	1.6	1.63
AT5G64000	19.92	21.92	21.46	11.61	9.68	9.42
AT1G53070	1.52	1.31	1.39	6.28	5.93	5.52
AT1G22570	5.95	7.13	6.45	2.69	2.21	2.23
AT3G04510	0.11	0	0.04	3.04	2.7	3.07
AT3G04630	9.45	10.46	8.83	21.38	21.34	20.12
AT1G05835	3.43	3.4	3.46	12.51	16.06	16.25
AT1G79840	0.46	0.54	0.39	1.93	1.99	1.89
AT1G13710	0.45	0.69	0.26	3.56	2.65	3.1
AT1G11210	18.23	17.58	17.03	8.27	8.62	7.45
AT5G07230	0	0	0	5.8	9.31	7.08
AT1G05575	40.78	36.65	33.06	11.32	12.11	12.52
AT4G16141	13.58	13.04	15.39	31.03	28.24	35.44
AT3G11980	0.02	0	0	1	0.88	0.98
AT1G30190	4.59	5.04	6.06	1.04	0.89	0.85
AT3G05470	0.4	0.32	0.31	1.79	1.85	1.51
AT5G10695	152.92	132.74	128.08	45.18	62.99	48.8
AT4G02800	0.9	0.96	1.19	4.41	4.01	4.61
AT4G22520	0.39	0.75	0.99	6.66	7.46	9.87
AT3G51290	0.2	0.29	0.25	1.36	1.33	1.44
AT4G34400	0.03	0.1	0.07	1.54	1.64	1.6
AT5G07010	6.25	8.2	7.11	16.3	15.27	16.01
AT3G14890	1.93	1.93	2.37	5.03	4.73	5.37
AT3G10870	1.96	2.35	2.19	6.6	7.59	7.91
AT5G50740	6.97	8.06	7.45	15.39	16.16	16.15
AT1G48100	4.73	5.35	5.74	11.82	10.57	11.14

AT5G50360	3.08	2.71	2.51	7.97	7.63	8.21
AT5G18020	0.75	1.39	1.28	9.32	11.85	9.66
AT1G01480	4.74	4.55	3.99	9.73	9.9	9.71
AT2G44830	3.07	3.52	3.64	7.05	6.69	7.73
AT4G09510	4.06	4.38	4.13	8.57	8.2	9.06
AT5G05410	6.97	7.5	6.78	2.07	2.36	2.69
AT2G38995	1.2	1.23	1.75	4.34	4.24	5
AT5G19890	12.73	11.5	11.02	5.21	3.74	4.34
AT1G02190	0.07	0.1	0.12	1.21	1.12	1.14
AT4G14330	0.44	0.28	0.3	1.52	1.45	1.62
AT2G39330	3.75	4.13	3.65	8.82	9.26	8.28
AT4G27570	0	0	0.02	1	1.27	1.4
AT4G14080	0	0	0	1.08	1.04	1.19
AT5G58120	10.82	10.98	12.21	5.99	4.66	6.57
AT1G62940	0.02	0	0	1.58	0.77	1.17
AT5G56530	4.33	4.65	5.18	10.37	9.43	10.41
AT5G26260	4.49	4.98	4.7	1.42	0.88	1.49
AT2G19910	0.02	0.05	0.01	0.5	0.91	0.98
AT5G62710	1.3	1.52	1.73	4.01	3.91	4.22
AT1G68160	9.48	11.06	12.31	21.66	23.65	23.53
AT5G46295	23.56	25.04	27.26	9.21	5.62	7.28
AT3G11430	0.6	0.38	0.27	2.19	2.2	2.59
AT4G00480	0.27	0.24	0.31	1.84	1.96	1.51
AT1G64640	10.46	13.38	10.69	27.62	30.58	26.13
AT3G54260	3.77	4.78	4.52	10.89	11.07	9.69
AT1G52410	1.61	1.87	1.95	4.26	3.93	4.3
AT4G29140	2.9	2.64	3.14	7.22	6.28	6.49
AT5G18030	1.04	1.01	1.32	9.99	10.33	8.21
AT1G29430	1.06	1.71	0.57	6.95	8.88	7.49
AT4G30170	5.66	5.22	4.91	0.64	1.53	1.41
AT1G29980	5.34	4.8	4.67	11.51	11.31	10.21
AT5G25090	1.11	1.44	1.06	7.09	5.25	6.45
AT4G29150	0.03	0.1	0.07	1.33	1.48	1.35
AT1G30730	6.39	5.99	6.09	2.39	2.72	2.98
AT5G51600	0.57	0.34	0.47	1.99	2.02	1.79
AT3G45930	25.97	28.77	27.57	52.71	65	59.99
AT5G53210	0.77	0.65	0.87	3.8	3.25	3.1
AT4G34850	0.03	0.07	0.09	1.44	1.51	1.29
AT3G55710	3.95	4.01	3.46	8.21	8.71	8.3
AT4G23750	5.37	5.2	4.36	11.7	10.87	11.03
AT1G64380	8.55	8.56	8.45	3.47	3.89	3.87
AT4G22130	2.39	2.96	3.12	6.75	5.75	6.34
AT1G43800	0.09	0.03	0.05	1.32	1.19	1.38

AT1G31040	0	0	0.04	1.92	3.32	1.76
AT1G44110	0.88	0.94	0.82	3.19	2.8	3.41
AT3G43850	3.37	3.26	3.07	0.43	0.58	0.64
AT1G17700	0.51	0.28	0.6	3.66	4.3	4.03
AT2G13610	3.09	3.36	2.89	6.37	6.4	6.4
AT1G08650	3.3	2.7	3.14	7.49	7.8	8.06
AT3G05936	25	27.59	29.45	8.95	10.99	13.5
AT5G11510	0.57	0.63	0.56	1.75	1.67	1.72
AT1G04520	5.43	6.14	6.58	13.34	11.88	12.84
AT4G10955	2.86	2.7	2.83	6.91	6.7	6.65
AT5G66750	1.58	1.44	1.5	3.59	3.54	3.4
AT5G45700	1.23	1.5	1.26	4.62	5.25	4.94
AT1G54200	1.6	1.71	1.85	4.76	5.09	6.14
AT1G07370	4.15	4.75	3.84	12.68	16.17	11.49
AT2G31160	2.52	2.85	3.58	9.04	8.92	8.37
AT5G58840	0.82	0.74	0.92	2.42	2.25	2.44
AT4G11211	57.71	54.63	46.45	138.92	164.25	132.79
AT3G24900	8.14	8.21	9.81	4.91	3.55	4.45
AT5G06150	1.38	1.19	1.34	4.96	4.37	3.72
AT4G03270	0.95	0.76	1.25	4.8	3.75	4.14
AT3G58650	0.25	0.19	0.33	1.28	1.18	1.22
AT2G40230	2.85	3.71	3.42	7.97	8.29	7.32
AT3G17680	0.89	0.89	0.7	3.61	2.84	3.87
AT1G02610	10.5	11.35	12.86	4.88	4.77	4.59
AT2G42990	0.03	0.11	0	1.11	1.6	1.67
AT3G44735	28.29	27.13	30.22	52.65	61.16	63.7
AT3G15270	1.4	1.19	1.24	6.57	5.65	5.41
AT5G64510	11.03	11.13	11.6	6.58	4.95	5.01
AT2G16440	2.3	2.04	2.5	4.48	4.61	4.94
AT1G73590	2.48	2.24	2.26	7.89	5.55	5.86
AT1G54385	0.56	0.46	0.44	1.82	2.44	2.72
AT2G36200	0.47	0.56	0.52	1.54	1.54	1.53
AT3G25900	2.43	1.71	2.84	6.4	6.64	6.96
AT5G51990	2.62	2.33	3.38	0.14	0.28	0.07
AT3G19390	4.18	3.27	4.05	0.75	0.93	1.4
AT2G25200	11.93	10.73	12.56	4.92	5.66	6.31
AT5G22460	1.9	1.54	1.86	6.92	4.8	6.1
AT1G04110	1.23	1.02	1.05	2.98	2.61	3.36
AT3G60900	0.76	0.6	0.66	2.75	2.61	2.63
AT5G55720	0.03	0	0	0.99	1.19	1.05
AT5G44540	0.29	0.11	0.53	4.04	5.06	4.76
AT3G52130	0.18	1.02	0.43	5.69	5.98	4.57
AT3G11480	0.52	0.71	0.6	2.77	2.66	2.55

AT1G78430	0.6	0.43	0.64	3.18	2.64	2.74
AT2G22840	2.26	1.97	2.4	6	5.07	5.02
AT3G61250	0.27	0.09	0.24	2.16	2.7	1.85
AT1G69230	3.33	3.34	2.28	10.34	12.63	12.16
AT3G57640	8.6	7.56	9.02	3.67	3.93	3.74
AT5G46960	1.52	1.75	1.6	5.82	6.68	6.8
AT1G67040	0.79	1	0.95	2.3	2.37	2.45
AT3G06030	0.89	0.84	0.67	2.55	2.25	2.61
AT4G21510	12.85	12.46	11.88	5.48	5.67	6.05
AT1G14440	1.73	2.08	2.51	6.72	6.72	10.09
AT2G29310	6.81	7.93	7.3	14.08	17.05	16.9
AT3G13650	1.54	1.78	2.03	6.83	6.08	6.37
AT3G54340	0	0	0.16	2.08	1.4	3
AT2G37390	0.18	0.49	0.35	3.11	2.46	2.56
AT5G01840	1.83	1.46	1	4.89	5.4	5.98
AT3G62740	0.07	0.05	0	1.41	0.71	1.1
AT3G02480	7.53	10.93	9.18	24.61	30.93	29.91
AT3G46490	0.48	0.28	0.6	2.58	2.4	2.22
AT3G11600	6.94	6.07	6.28	15.9	17.56	16.55
AT1G09575	8.39	7.59	8.52	3.53	3.14	3.9
AT3G27400	4.07	3.53	4.25	9.15	7.62	8.53
AT4G27230	44.42	42.62	36.11	83.79	91.45	75.53
AT5G02760	6.6	4.89	6.39	13.77	11.5	13.44
AT1G06100	0	0	0	1.35	1.56	1.16
AT1G77270	0.56	0.32	0.4	1.52	1.8	1.93
AT2G14900	4.24	5.4	4.9	13.2	17.43	17.04
AT1G68110	3.77	3.57	3.07	7.49	7.46	7.77
AT3G26932	1.62	1.37	1.86	4.59	4.46	4.34
AT3G50410	2.04	1.66	1.5	5.41	6.62	5.75
AT3G51970	9.74	8.17	9.33	4.16	4.14	4.41
AT5G44550	4.72	4.39	4.61	10.45	11.88	14.11
AT5G65390	5.46	5.07	5.45	14.32	14.15	13.21
AT2G29890	0.48	0.48	0.64	1.58	1.52	1.85
AT1G72670	0.47	0.77	0.57	2.38	3	2.49
AT1G20350	12.6	12.03	11.56	4.95	5.62	5.8
AT2G40820	1.92	2.12	1.89	4.43	5.35	4.69
AT4G13210	0.16	0.06	0.14	1.25	1.1	1.37
AT3G56100	0.18	0.1	0.16	1.04	1.36	1.23
AT5G50790	0.28	0.31	0.19	1.95	1.99	2.41
AT5G07280	0.29	0.2	0.23	0.88	0.87	0.92
AT5G07690	5.21	5.2	5.26	10.99	9.84	13.24
AT5G37010	0.85	0.88	0.85	2.59	2.17	2.56
AT4G30130	0.14	0.15	0.17	1.04	0.81	1.04

AT1G18370	0.4	0.24	0.43	1.38	1.28	1.19
AT5G43580	7.27	7.42	10.22	20.7	21.18	25.88
AT5G62550	1.35	1.34	1.15	3.21	3.42	3.64
AT3G20670	15.01	15.57	12.17	32.58	42.86	33.42
AT3G07255	0.51	0.7	0.55	3.8	2.44	3.13
AT3G55660	0.34	0.51	0.51	1.61	1.98	1.85
AT4G34900	0.45	0.37	0.44	1.18	1.09	1.16
AT5G55340	0.07	0.08	0	1.02	1.17	1.81
AT3G25110	2.89	2.64	2.77	6.01	6.09	6.47
AT4G05190	0.71	0.5	0.56	1.81	1.69	1.84
AT3G27660	0.12	0.07	0	2.02	2.43	1.74
AT5G57123	0	0.56	0.27	4.52	7.56	7.24
AT4G33467	4.37	6.33	5.36	14.91	14.37	17.11
AT4G21590	0.16	0.09	0.06	1.37	1.74	1.67
AT1G63650	0.14	0.21	0.17	0.98	1.15	1.3
AT3G61750	3.18	2.4	3.26	8.43	7.03	6.65
AT1G50110	1.07	0.68	0.76	2.9	3.66	3.2
AT4G25240	0.49	0.5	0.41	1.75	1.58	2.13
AT5G13520	2.88	2.94	3.11	6.37	6.34	5.46
AT5G26670	2.57	2.48	2.47	5.23	5.98	6.89
AT2G25270	1.31	1.73	1.52	3.49	3.71	3.93
AT1G21090	2.72	2.76	1.92	7.11	6.48	6.99
AT2G13820	5.24	5.28	5.46	13.69	12.47	11.23
AT3G01190	4.77	3.89	4.07	1.41	1.16	1.28
AT3G06035	6.32	8.08	7.2	14.4	16.43	15.64
AT1G47510	3.65	2.72	3.14	7.81	6.67	8.67
AT2G29110	5.51	4.73	5.65	3	2.2	2.86
AT2G41800	0.81	0.65	0.73	3.23	3.06	2.44
AT2G19170	1.64	1.79	1.5	3.96	3.19	4.24
AT3G44550	2.04	1.72	1.55	4.95	4.11	4.34
AT5G36970	1.81	2.16	1.85	5.65	5.19	5.8
AT3G57830	1.16	0.69	1.16	3.48	3.46	2.7
AT3G63430	0.51	0.09	0.35	1.8	1.79	1.54
AT1G54020	0.06	0.03	0.07	1.26	1.42	0.85
AT3G03820	0.83	1.19	1.03	6.83	6.74	6.06
AT5G60930	0.43	0.51	0.41	1.14	1.14	1.27
AT1G79420	0.66	0.7	0.84	2.55	2.87	2.43
AT5G44635	1.12	1.09	1.22	2.58	2.38	2.63
AT5G67270	0.91	0.97	0.85	3.38	2.83	3.46
AT3G46320	27.85	32.33	26.7	57.84	70.54	56.03
AT1G64450	1.41	1.57	1.21	4.24	3.72	4.17
AT5G20240	0.37	0.29	0.25	2.56	2.04	3.3
AT1G80160	2.85	3.54	3.08	7.44	10.29	9.4

AT4G29240	1.44	1.6	1.52	3.85	3.71	4.62
AT2G32200	19.2	23.59	19.16	7.79	8.21	8.66
AT4G15620	10.09	8.03	7.53	18.96	17.29	18.62
AT5G55520	0.3	0.29	0.3	1.06	1.18	1.2
AT1G26945	4.01	5.79	8.19	18.38	19.67	19.22
AT5G51590	1.46	1.4	1.31	3.77	3.56	3.55
AT2G42570	3.04	2.99	3.61	6.94	6.35	7.08
AT4G08780	4.22	4.72	5.58	1.4	1.75	1.92
AT5G25475	1.85	2.24	2	5.27	6.33	5.08
AT5G11590	2.63	2.43	2.52	7.03	6.05	6.56
AT3G57920	0.29	0.22	0.15	1.46	1.49	2.06
AT2G46780	2.26	3.11	3.27	6.67	6.47	6.83
AT1G03780	0.38	0.42	0.37	1.32	1.21	1.27
AT2G45480	0	0.09	0.06	0.91	0.73	1.03
AT1G03820	7.35	8.71	9.94	16.24	18.09	20.36
AT4G18350	0.18	0.11	0.05	0.83	0.88	1.03
AT4G30290	6.97	6.67	7.38	3.55	2.91	2.57
AT3G60120	1	0.76	1.3	3.83	2.97	2.96
AT1G65710	0.66	0.31	0.24	2.23	1.78	1.91
AT5G06940	0.12	0.01	0.05	0.52	0.54	0.73
AT1G50240	0.38	0.33	0.42	1.05	0.95	1.25
AT4G15390	3.82	3.38	4.17	1.41	1.6	1.46
AT4G14550	1.67	1.27	1.98	4.81	5.95	4.83
AT1G13670	0.15	0.45	0.28	2.07	2.27	2.53
AT5G66740	1.34	1.62	1.73	4.97	3.9	4.11
AT3G13175	2.7	3.47	2.11	11.33	11.79	8.75
AT5G09530	1.93	1.96	1.54	5.19	5.37	4.28
AT5G58860	0.7	0.8	0.86	2.14	2.58	2.44
AT1G34245	0.19	0.21	0.15	3.61	2.57	3.28
AT5G65690	1.07	0.81	1.04	0.16	0.2	0.16
AT3G54180	1.12	1.57	1.81	5.01	3.92	4.71
AT2G25880	0.82	0.8	0.9	3.33	2.49	4.13
AT4G15830	1.4	1.33	1.58	4.08	4.98	4.12
AT3G02310	0.04	0.1	0.14	2.29	1.91	1.03
AT5G64110	0.28	0.35	0.49	3.08	1.74	2.16
AT4G24110	9.56	8.71	7.93	4.25	3.23	3.42
AT5G60490	1.71	1.33	1.3	4.2	4.36	5.02
AT5G28630	15.26	20.76	19	8.03	8.26	7.25
AT1G20310	8.67	9.92	7.99	3.49	3.75	3.82
AT5G36910	8.89	12.6	10.18	22.04	25.27	22.7
AT1G30100	0.29	0.46	0.48	1.4	1.5	1.86
AT3G26510	54.13	49.76	39.37	25.74	24.06	22.43
AT2G35310	0.12	0.09	0.03	1.29	1.4	1.15

AT5G17160	0.77	0.92	0.79	2.87	2.76	2.09
AT2G42350	11.22	11.44	11.41	5.19	6.43	5.1
AT3G06740	5.39	8.18	6.57	18.43	14.95	15.66
AT5G38940	1.68	2.01	1.79	5.67	4.46	5.57
AT4G28310	6.88	6.93	6.15	13.62	14.5	12.75
AT5G38120	1.18	1.13	1.14	2.88	3.03	2.66
AT1G26100	2.09	2.21	1.83	5.12	5.52	6.23
AT1G68640	0.13	0	0.08	1.18	0.79	0.77
AT4G32830	1.17	1.39	1.87	4.82	5.02	3.98
AT4G35060	2.55	1.91	2.23	6.93	8.5	6.7
AT2G20750	4.07	3.67	4.04	11.69	7.87	9.23
AT2G31010	0.68	0.64	0.82	1.88	1.67	2.09
AT3G18960	0.77	1.02	1.44	5.02	4.42	3.59
AT5G18080	1.14	1.4	1.19	6.04	6.33	9.07
AT5G43990	0.58	0.48	0.58	1.48	1.49	1.69
AT1G49450	1.76	1.68	2.18	4.01	4.03	4.47
AT1G11925	0.25	0.27	0.45	2.98	2.76	3.49
AT1G73620	0.52	0.58	0.82	2.5	3.47	2.76
AT5G62230	0.1	0.13	0.08	0.6	0.58	0.59
AT1G68450	0.6	0.58	0.53	3.06	3.46	4.25
AT1G02630	0.06	0.07	0.02	1.16	1.08	0.61
AT1G75640	0.37	0.24	0.35	1.03	0.86	1.22
AT5G43890	0.98	1.02	0.89	0.08	0.07	0.07
AT1G55990	0.66	0.82	0.71	3.93	3.78	4.31
AT1G74460	0.6	0.77	0.64	1.98	3.06	2.9
AT4G05520	0.95	0.91	0.66	2.16	2.36	2.73
AT1G24260	0.46	0.41	0.47	2.25	3.28	2.21
AT2G24700	0.04	0.02	0.02	0.49	0.67	0.6
AT1G46264	2.78	3.3	3.19	6.39	5.85	7.23
AT3G23670	0.22	0.3	0.24	0.95	0.71	0.8
AT5G28290	1.81	1.91	2	4.12	3.89	3.66
AT5G15120	0.39	0.78	0.77	2.7	2.97	2.38
AT3G05950	5.97	5.5	6.68	2.53	2.17	2.49
AT5G13840	1.17	0.85	1.05	2.72	3.01	2.6
AT5G09876	11.43	10.86	12.71	4.58	2.94	5.09
AT1G60270	1.03	1.14	1.52	3.66	3.2	3.26
AT3G21950	2.04	1.37	2.07	4.07	4.89	5.03
AT3G55840	6.99	5.39	5.76	2.11	2.98	2.55
AT3G54580	0.65	0.65	0.66	0.12	0.1	0.18
AT5G67080	1.81	1.56	2.02	4.83	4.11	4.28
AT5G03390	2.75	1.95	2.13	5.15	4.87	5.56
AT1G29450	0.89	0.27	0.45	3.99	3.73	3.13
AT3G23740	0.45	0.5	0.62	2	1.7	1.54

AT5G59090	1.16	1.41	1.34	0.29	0.53	0.3
AT5G48600	0.5	0.62	0.65	1.55	1.26	1.35
AT1G09350	0.34	0.23	0.3	1.44	1.71	1.65
AT3G51280	0.67	0.86	0.9	2.48	2.39	2.28
AT3G61950	0.84	0.43	0.52	1.94	2.39	2.48
AT4G21970	0.88	0.57	0.29	2.87	3.94	3.62
AT4G13820	2.63	2.59	3.23	1.46	1.08	1.28
AT5G25210	7.75	8.99	8.46	3.82	3.82	4.25
AT3G55700	1.93	1.94	2.22	0.53	0.74	0.69
AT1G74890	5.69	7.35	7.95	15.55	14.15	13.19
AT5G61160	0.08	0.25	0.14	0.86	1.1	1.47
AT1G19540	0.84	0.6	0.71	2.24	2.55	2.8
AT3G23010	2.67	2.79	3.11	1.41	1.31	1.28
AT5G57785	9.05	7.08	7.09	21.2	23.36	17.81
AT1G15890	2.1	2.52	2.09	0.95	1.1	0.82
AT1G29510	0.32	0.62	0.5	4.04	2.92	2.87
AT3G49750	1.05	0.98	1.41	4.65	3.06	3.87
AT1G80240	2.21	1.86	1.95	4.05	5.5	5.43
AT5G03870	0.54	0.37	0.52	1.68	1.86	1.94
AT4G22560	1.48	1.06	1.81	4.33	6.64	4.61
AT4G20420	0.32	0.63	0.57	4.18	4.36	2.44
AT5G48650	0.15	0	0.02	0.63	0.75	0.83
AT4G07995	51.3	53.03	62.64	17.37	19.04	28.81
AT3G16410	0.71	0.62	0.67	1.9	1.73	2.79
AT5G22545	8.76	9.82	11.87	3.18	3.48	3.69
AT5G08020	1.11	1.18	1.08	2.51	2.42	2.57
AT2G32487	13.78	13.35	16.76	5.47	6.74	6.27
AT2G19970	0.26	0.29	0.56	2.45	2.8	2.41
AT4G33260	0.55	0.5	0.55	1.9	1.49	2.21
AT5G28237	0.07	0.2	0.11	1	0.75	0.92
AT3G03130	0.66	0.52	0.56	2.27	1.55	1.86
AT2G19780	2.55	2	2.56	4.73	4.72	5.48
AT3G12870	1.23	1.3	1.31	4.06	3.76	4.3
AT1G64940	0.65	0.65	0.71	2.02	2.04	1.77
AT3G10310	0.19	0.22	0.23	0.92	0.69	0.72
AT4G38080	4.29	5.25	5.33	11.53	11.35	11.08
AT4G13710	0.76	0.79	1.09	3.02	2.28	2.39
AT2G41050	1.01	1.22	1.12	2.8	3.06	2.91
AT5G20635	1.26	0.91	1.09	3.22	3.81	3.22
AT1G71050	10.03	7.77	7.09	16.28	17.71	19.61
AT1G01110	0.48	0.27	0.36	1.26	1.59	1.38
AT5G09300	0.42	0.49	0.63	1.77	1.83	1.47
AT1G49900	1.36	0.95	1.08	0.39	0.34	0.29

AT1G79900	2.06	1.55	1.83	4.79	4.04	4.54
AT4G26660	0.37	0.33	0.39	0.96	1.24	1.22
AT3G58550	3.05	2.87	3.3	7.18	8.05	7.14
AT4G29690	0.78	1.14	1.11	0.14	0.14	0.14
AT5G11160	2.56	2.96	4.36	8.48	7.43	9.3
AT4G01580	0.91	1.14	0.85	3.39	4.4	3.49
AT5G56220	1.09	0.89	1.15	1.99	2.64	2.35
AT3G56220	0.2	0.23	0.11	1.63	2.4	1.97
AT5G46690	4.16	3.98	2.92	7.99	7.15	8.08
AT1G47980	1.24	0.89	1.35	3.38	4.29	3.12
AT2G32765	4.45	5.16	5.23	10.98	13.72	12.24
AT3G57060	0.6	0.57	0.66	1.4	1.12	1.39
AT3G63280	0.89	0.9	0.77	1.86	2.48	2.32
AT4G04460	1.25	1.26	1.17	3.34	2.85	2.59
AT2G01210	0.6	0.46	0.59	1.44	1.39	1.53
AT2G04032	0.44	0.31	0.27	1.37	1.53	1.82
AT3G28345	0.83	0.69	0.7	1.47	1.56	1.5
AT5G23400	1.55	1.21	1.49	2.93	2.93	2.93
AT1G30760	2.96	3.44	2.83	0.99	1.21	1.64
AT3G53160	0.8	0.76	1.07	2.11	2.25	2.59
AT2G07170	0.22	0.3	0.33	1.26	1.12	0.79
AT1G14480	3.53	3.5	3.95	1.87	1.83	1.44
AT5G63580	1.32	1.91	1.57	3.88	4.17	4.81
AT1G23000	0.5	0.61	0.69	2.28	2.23	1.63
AT2G25980	1.46	1.02	1.41	0.22	0.35	0.25
AT2G33400	1.78	1.73	2.45	5.86	4.34	5.18
AT3G14190	1.31	1.25	1.45	4.42	4.17	3.85
AT2G37870	2.09	2.31	2.65	6.69	8.46	7.12
AT4G39480	0.07	0.1	0.14	0.84	0.63	0.8
AT4G21270	0.25	0.4	0.6	1.75	1.24	1.4
AT4G17000	0.24	0.17	0.35	1.17	0.9	0.92
AT5G02550	2.7	2.32	2.58	8.19	10.32	8.66
AT5G12050	3.12	2.04	3.31	5.88	6.01	7.22
AT4G13540	1.26	0.79	0.77	2.99	3.25	4.36
AT5G63920	0.7	0.76	0.77	2.09	2.03	1.46
AT3G43960	0.4	0.47	0.6	1.8	1.74	1.73
AT5G05240	1.09	0.53	0.95	2.25	2.23	2.63
AT1G78120	0.82	0.88	0.98	2.16	1.97	2.42
AT1G29500	0.85	0.85	0.67	4.04	3.55	3.62
AT1G51460	0.34	0.81	0.53	1.88	2.06	1.57
AT4G19030	1.61	0.97	1.18	0.1	0.2	0.1
AT4G35730	1.41	1.34	1.67	3.91	3.07	3.13
AT4G37810	0.81	1.19	1.05	3.89	3.98	6.52

AT1G44830	1.09	1.15	1.54	5.57	3.09	5.24
AT3G02885	1.13	1.25	0.88	5.37	5.17	4.65
AT5G10080	0.26	0.36	0.24	1.56	0.94	1.2
AT5G19340	1.27	0.87	0.82	2.82	3.24	3.19
AT4G28940	1.29	1.61	2	4.31	4.95	3.64
AT5G03680	0.47	0.28	0.44	1.2	1.21	1.45
AT2G26180	1	0.89	1	2.56	2.17	2.74
AT2G22510	1.94	1.84	2.1	8.54	5.48	6.22
AT1G44970	0.2	0.26	0.34	1.86	1.08	1.46
AT3G19270	1.07	1.07	1.06	2.55	2.75	2.33
AT2G28410	6.17	6.17	5.38	12.96	17.99	13.28
AT5G44560	1.97	1.52	2.48	4.56	4.88	5.48
AT2G22420	1.68	1.59	1.48	3.33	4.3	3.99
AT3G19050	0.07	0.09	0.11	0.25	0.31	0.29
AT1G75590	0.45	0.49	0.29	3.13	2.11	2.56
AT2G18370	2.86	2.19	3.01	8.15	6.79	7.87
AT3G14760	3	3.33	3.05	7.65	7	6.95
AT5G40330	0.79	0.58	0.62	2.72	3.19	2.24
AT1G53700	0.63	0.46	0.47	1.42	1.67	1.8
AT4G07960	0.36	0.46	0.4	1.32	1.4	1.02
AT1G23340	0.22	0.24	0.3	1.22	1.06	1.2
AT4G14200	1.41	0.86	1.14	2.27	2.86	3.18
AT5G53486	4.37	5.06	5.99	10.84	12.32	11.87
AT4G01670	7.71	6.7	6.26	3.1	2.68	3.68
AT1G60060	0.94	1.37	1.13	3.17	2.54	2.91
AT2G24490	3.3	2.88	3.42	6.93	6.84	5.84
AT4G18640	1.41	2	1.65	4.05	3.07	3.44
AT5G51440	6.73	6.73	7.97	2.99	3.4	3.76
AT3G25490	0.24	0.38	0.21	1.38	1.04	1.24
AT3G48340	1.18	1.02	1.47	0.13	0.22	0.26
AT1G67400	1.23	1.13	1.03	2.81	3.09	4.17
AT3G24240	0.41	0.65	0.33	0.08	0.08	0.03
AT2G30370	0.19	0.16	0.23	1.61	1.23	1.52
AT5G19520	0.57	0.55	0.37	0.09	0	0.09
AT2G35730	2.59	2.68	2.87	6.52	7.61	7.38
AT2G31270	1.96	1.94	1.93	3.73	3.51	5.22
AT4G31730	6.02	4.99	6.09	10.37	14.2	12.38
AT1G30600	1.5	1.29	1.36	3.03	3.29	2.38
AT1G30650	0.46	0.21	0.34	1.2	1.55	1.25
AT4G01730	0.52	0.63	0.55	1.46	1.56	1.78
AT3G14550	0.32	0.25	0.3	1.43	1.29	1.19
AT2G30820	0.38	0.33	0.56	1.69	1.29	1.54
AT1G21326	4.09	4.79	4.41	2.02	1.69	1.79

AT3G60890	0.11	0	0.08	0.76	3.78	3.12
AT5G12970	0.18	0.08	0.19	0.78	0.49	0.85
AT5G57090	0.82	0.55	0.52	0.05	0.12	0.1
AT5G48820	0.62	0.42	0.23	1.74	2.07	2.17
AT5G25810	0.26	0.29	0.33	1.7	1.71	1.88
AT3G29590	1.23	0.91	1.27	2.55	2.67	2.65
AT3G53600	1.05	1.38	0.67	2.85	5.22	4.96
AT5G45960	0.68	0.58	0.58	1.81	2.4	1.73
AT1G58300	6.95	8.1	6.81	3.02	2.69	4.53
AT2G47230	0.5	0.43	0.4	1.26	1.19	1.13
AT3G57760	2.47	2.5	2.77	1.02	0.95	1.21
AT4G13370	0.86	0.7	0.64	1.7	1.69	1.67
AT1G10780	1.22	0.63	0.7	2.66	2.31	2.32
AT5G26660	0.35	0.49	0.52	1.72	1.45	1.65
AT4G21760	1.1	0.84	1.19	3.25	2.45	2.23
AT2G22610	0.18	0.22	0.15	0.54	0.6	0.7
AT2G38620	0.62	0.92	0.76	1.88	2.46	2.65
AT4G34770	3.85	2.44	2.93	7.39	8.6	9.37
AT2G18470	0.11	0.02	0.1	0.52	0.54	0.46
AT5G15600	4.96	5.99	4.45	12	11.56	10.17
AT1G72250	0.55	0.43	0.48	1.18	0.96	1.08
AT5G65170	0.95	1.02	0.82	2.85	2.45	2.19
AT5G27550	0.72	0.73	1.19	2.11	2.14	1.97
AT2G20515	1.11	0.9	0.93	3.84	3.2	3.56
AT2G16580	0.85	0.35	0.41	2.82	3.43	3.79
AT3G02120	3.82	5.34	2.71	9.42	12.39	11.13
AT4G11140	0.64	0.67	0.79	1.97	2.87	2.21
AT1G22030	2.38	2.33	2.33	5.86	3.96	6.46
AT1G69920	3.03	3.41	2.66	6.47	5.44	7.22
AT3G20150	0.35	0.22	0.34	0.81	0.72	0.85
AT2G38160	0.14	0.19	0.32	1.11	1.26	1.14
AT4G16970	0.65	0.6	0.66	1.31	1.31	1.46
AT3G43190	0.46	0.43	0.35	1.14	1.35	0.94
AT1G20930	2.05	2.27	2.03	3.99	4.44	4.62
AT3G22760	0.81	0.86	1.02	1.7	2.4	2.52
AT5G40730	8.08	10.78	10.08	17.79	22.25	23.81
AT5G23910	0.52	0.65	0.76	1.47	1.46	2.18
AT5G39180	1.51	3.69	6.52	0.29	0.56	0.21
AT4G36570	4.3	4.77	3.99	10.41	17.16	13.72
AT1G16630	0.42	0.29	0.45	1.05	0.9	1.11
AT3G61610	1.85	2.01	1.79	4.11	5.43	3.84
AT5G23830	5.6	5.04	6.3	2.94	1.98	1.54
AT4G29610	2.63	2.74	1.91	0.55	0.64	0.92

AT4G15330	0.7	0.87	0.88	0.13	0.24	0.12
AT1G78260	2.37	2.71	1.92	5.69	5.79	4.4
AT5G39110	6.11	5.62	6.08	2.47	3.42	2.71
AT4G37490	0.4	0.3	0.53	1.32	1.2	1.78
AT1G14490	0.1	0.23	0.4	1.93	1.59	1.12
AT1G68200	1.24	1.17	1.14	3.02	2.71	2.86
AT5G56120	1.85	1.13	1.18	3.48	3.68	3.89
AT4G25150	0.97	0.88	1	2.79	2.39	2.92
AT1G15760	1.42	1.76	1.83	5.02	5.06	3.52
AT2G37380	0.86	0.99	1.21	2.36	2.61	2.91
AT1G80690	4.18	3.09	5.38	8.02	9.73	9.51
AT1G15040	2.81	2.81	3.06	1.11	1.33	1.69
AT1G13600	0.7	0.58	0.55	1.97	2.53	2.74
AT2G29125	0.8	1.32	0.7	3.35	4.03	4.38
AT2G19590	0.79	0.91	1.18	2.34	2.4	2.58
AT1G65390	2.2	2.47	2.37	0.88	1.17	1.05
AT1G29460	0.8	0.62	0.34	1.82	2.75	3.06
AT5G66800	0.75	0.63	0.93	2.64	2.96	2.67
AT5G50335	2.62	2.73	3.14	7.55	12.46	8.26
AT3G48630	7.34	8.13	8.27	4.14	3.62	2.83
AT2G45430	1.78	2.29	2.16	0.81	0.69	0.6
AT1G27880	0.4	0.49	0.51	1.08	1.06	1.01
AT5G53820	1.53	2.44	1.86	6.9	8.25	6.54
AT5G45850	0.31	0.32	0.28	1.2	1.64	0.85
AT3G54000	3.81	3.58	3.28	2.02	1.66	1.52
AT1G65900	1.07	0.66	0.82	1.82	2.25	2.56
AT3G02930	0.41	0.32	0.49	0.94	1.06	1.04
AT2G28690	0.45	0.28	0.47	1.53	2.42	1.51
AT1G13609	7.08	7	8.12	3.19	1.71	3.13
AT3G12970	0.36	0.37	0.45	1.27	1.35	1.37
AT1G31320	1.73	2.51	3.19	5.61	6.3	5.78
AT2G37560	0.6	0.91	0.7	2.04	1.93	1.88
AT2G33793	0.27	0.42	0.34	1.82	2.05	1.27
AT5G37000	0.35	0.3	0.21	0.96	0.9	0.97
AT5G07800	0.88	0.72	0.71	2.17	1.49	2.76
AT5G50010	0.52	0.49	0.26	1.3	1.45	2.19
AT5G41830	2.6	2.66	3.21	1.29	1.03	1.79
AT4G21820	0.08	0.13	0.1	0.45	0.29	0.41
AT3G02500	0.86	0.43	0.8	1.75	2.38	2.31
AT3G01410	2.62	1.69	1.96	4.99	4.54	4.09
AT1G17960	3.52	2.65	3.53	1.9	1.19	1.49
AT3G25980	1.86	2.3	2.05	4.67	4.05	5.11
AT1G62870	0.76	0.89	0.97	1.53	1.82	2.1

AT3G21090	0.15	0.26	0.24	0.84	0.83	0.6
AT1G76740	0.07	0.06	0.06	0.25	0.25	0.27
AT2G15880	0.73	0.63	0.66	0.2	0.15	0.24
AT3G02030	1.09	0.81	0.83	2.04	1.66	2
AT1G09450	0.15	0.06	0.35	0.89	0.67	0.77
AT5G01910	0.78	0.49	0.52	2.18	2.18	2.14
AT2G38070	0.56	0.58	0.82	1.56	1.63	1.38
AT3G13275	11.05	12.24	11.11	4.39	5.79	3.86
AT1G62760	0.52	0.82	0.43	1.96	1.89	1.62
AT1G11120	0.55	0.48	1.01	1.89	3.4	2.79
AT2G32590	0.62	0.63	0.48	1.56	1.9	1.16
AT2G42900	1.17	1.15	1.14	3.74	3.09	2.35
AT3G22880	0.83	0.51	0.65	1.95	2.19	1.59
AT2G41340	0.42	0.87	0.47	2.14	2.13	1.79
AT3G05400	0.17	0.22	0.14	0.91	0.67	0.82
AT5G57760	6.87	7.93	8.93	13.92	16.76	18.49
AT1G73360	0.45	0.48	0.49	1.48	1.1	1.01
AT1G64625	0.15	0.33	0.2	0.74	0.98	0.82
AT1G26540	0.58	0.46	0.61	1.37	1.16	1.25
AT2G36026	1.57	1.6	1.47	4.13	3.81	3.8
AT1G12845	3.85	4.05	3.54	9.16	8.31	7.54
AT5G40000	1.49	1.47	1.65	0.82	0.56	0.24
AT3G05980	0.19	0.57	0.15	1.12	2.03	1.42
AT4G11190	1.06	1.04	1.17	2.88	3.28	3.18
AT2G18500	0.69	0.49	0.52	2.2	1.48	1.66
AT4G38850	1.67	1.14	1.71	4.67	6.23	4.94
AT5G27630	0.16	0.13	0.28	0.76	0.55	0.74
AT4G15248	6.75	5.96	4.91	10.28	14.65	13.45
AT5G56840	2.27	1.97	2.82	5.18	4.33	5.29
AT5G56960	0.45	0.45	0.52	1.45	1.54	1.01
AT5G24330	0.43	0.29	0.49	1.29	1.25	1.5
AT3G50870	0.43	0.43	0.24	1.2	1.79	1.34
AT5G55820	0.12	0.11	0.2	0.48	0.35	0.37
AT1G35290	0.36	0.61	0.38	2.21	1.64	2.01
AT3G11000	0.25	0.17	0.25	0.85	0.59	0.89
AT5G48310	0.16	0.15	0.19	0.49	0.4	0.6
AT4G11170	0.29	0.36	0.47	1	0.8	0.81
AT2G43610	4.05	3.08	3.37	1.72	1.44	1.58
AT1G49200	2.9	2.14	2.07	4.35	5.63	5.53
AT2G25460	2.54	2.33	2.15	1.46	1.04	1
AT2G15440	0.87	1.12	0.88	2.11	2.12	2.93
AT2G46640	1.75	1.51	1.61	3.81	3.88	2.94
AT4G13990	0.97	1	1.13	2.57	2	1.95

AT1G52750	0.42	0.5	0.41	1.05	1.01	1.25
AT1G50745	0.59	1.03	0.95	0.2	0.08	0.23
AT4G24010	0.18	0.08	0.13	0.36	0.55	0.71
AT3G17360	0.12	0.1	0.08	0.33	0.26	0.25
AT3G48350	1.4	1.16	1.29	2.72	2.86	2.42
AT4G22810	0.99	1.42	1.34	0.2	0.34	0.44
AT2G27990	0.3	0.26	0.37	0.88	0.85	0.97
AT5G44350	2.45	2.1	3.05	1.16	0.42	1.23
AT1G63480	1.24	1.59	2.22	3.62	3.87	3.38
AT2G45900	0.46	0.18	0.21	0.81	0.93	0.86
AT4G37030	0.79	0.79	0.7	1.62	1.5	1.64
AT3G57860	0.95	0.42	0.37	1.99	2.11	1.76
AT1G47130	29.68	25.94	20.67	13.17	13.8	9.53
AT3G57200	0.15	0.24	0.15	0.76	0.68	0.66
AT1G80370	0.2	0.28	0.72	1.23	2.23	1.48
AT2G06925	5.08	4.08	6.18	10.32	13.72	9.12
AT1G53140	0.4	0.41	0.5	1.09	1.1	0.83
AT5G54400	0.43	0.31	0.43	1.32	1.38	1.41
AT1G20990	0.25	0.32	0.2	0.76	1.27	1.24
AT1G34355	0.13	0.17	0.18	0.44	0.34	0.54
AT4G12620	0.78	0.93	0.99	1.49	1.76	2.34
AT3G16150	1.66	1.18	1.47	3.52	2.77	2.92
AT2G44190	0.41	0.22	0.32	1.02	0.79	1.27
AT2G33560	0.41	0.48	0.34	0.98	1.33	1.48
AT2G45050	1.57	1.83	1.81	3.84	3.17	3.83
AT5G59740	0.67	0.82	0.92	2.06	1.72	2.07
AT1G19530	8.7	7.69	8.2	4.8	3.7	3.63
AT2G42260	1.6	2.02	1.91	4.42	3.53	3.54
AT5G45810	0.33	0.48	0.52	1.04	1.19	1.41
AT1G56540	0.98	1.12	1.39	0.63	0.6	0.54
AT2G37300	1.71	1.95	1.56	2.99	4.13	5.1
AT3G59900	1.15	1.66	1.31	3.83	4.76	3.39
AT3G21305	6.99	10.09	9.52	14.47	19.67	20.89
AT3G52540	1.43	1.4	1.44	3.14	3.08	2.81
AT2G13550	0.94	0.61	0.87	2.25	2.39	2.36
AT4G39630	0.99	0.55	1.31	2.28	2.8	2.71
AT5G19170	0.68	0.82	0.67	1.44	1.87	1.9
AT2G18480	0.7	0.93	0.71	1.59	1.53	1.93
AT4G10630	0.59	0.65	0.65	1.25	2.23	1.9
AT2G44910	0.58	1	1.19	1.98	2.34	2.49
AT5G52300	1.28	1.11	1.49	0.47	0.68	0.61
AT3G59200	0.13	0.07	0.21	0.43	0.66	0.7
AT4G25760	0.89	0.89	0.56	1.99	3.95	2.93

AT4G34970	1.06	0.36	0.76	2.85	2.52	2.46
AT1G68330	0.82	0.38	0.57	1.92	2.08	1.42
AT4G28950	0.55	0.79	1.03	2.7	1.63	3.33
AT1G48330	3.34	3.2	2.86	7.45	9.02	7.31
AT3G06370	0.83	0.63	0.62	1.6	1.43	1.43
AT4G35420	1.41	1.09	1.08	2.27	2.67	2.72
AT3G24020	1.42	2.1	2.45	0.53	0.89	0.39
AT2G17036	1.85	1.55	1.45	0.72	0.58	0.71
AT5G44585	9.06	9.39	11.1	4.71	3.55	5.43
AT3G45610	1.97	1.66	1.95	3.81	3.23	4.78
AT5G65420	0.32	0.39	0.2	1.41	0.92	0.93
AT1G74420	0.14	0.11	0.18	0.75	0.41	0.59
AT5G49120	0.99	0.59	0.78	2.68	2.17	2.94
AT5G10970	0.68	0.84	0.63	2.31	2.11	1.51
AT5G65510	0.25	0.2	0.23	0.58	1.02	0.79
AT2G48130	0.82	0.56	0.79	2.55	2.2	1.98
AT1G75945	2.61	2.06	2.33	6.78	8.29	6.15
AT2G28710	1.62	1.63	1.67	4.43	3.67	3.64
AT2G28620	0.28	0.27	0.29	0.74	0.94	0.5
AT1G63100	0.21	0.21	0.47	0.79	0.9	0.82
AT1G17920	0.5	0.48	0.91	1.81	1.2	1.57
AT2G23540	0.92	0.92	1.26	2.29	1.89	2.37
AT2G03360	0.25	0.2	0.2	0.74	0.99	0.63
AT5G56370	2.35	1.97	2.47	1.13	0.84	1.45
AT4G02650	0.19	0.23	0.16	0.63	0.53	0.7
AT5G07810	0.17	0.12	0.2	0.65	0.36	0.41
AT1G11420	0.31	0.23	0.22	0.94	0.54	0.81
AT1G09812	1.61	1.78	1.75	5.01	3.62	4.31
AT3G22560	1.84	1.89	1.95	4.32	4.69	3.52
AT5G01490	0.25	0.28	0.44	1.1	0.89	0.9
AT4G14150	0.26	0.23	0.24	0.51	0.51	0.59
AT5G22530	5.69	5.94	5.75	1.73	2.78	3.63
AT4G30370	4.37	3.97	4.09	1.46	2.38	1.88
AT5G06590	0.59	1.15	0.9	1.85	2.25	2.1
AT1G09000	0.2	0.49	0.28	0.86	1.06	0.75
AT4G04810	3.78	4.26	4.15	2.6	1.39	1.23
AT2G25220	0.25	0.28	0.57	0.99	0.95	1.13
AT5G45730	0.19	0.16	0.16	0.66	0.61	0.51
AT4G25540	0.09	0.06	0.13	0.33	0.33	0.29
AT1G78940	0.24	0.08	0.16	0.57	0.38	0.68
AT3G26512	6.58	6.93	5.41	1.81	2.33	3.71
AT5G66580	2.13	1.55	1.73	3.81	5.56	3.74
AT1G23760	1.46	1.64	1.29	0.55	0.78	0.82

AT1G56020	1.68	0.9	1.09	3.08	2.54	2.39
AT1G01690	0.74	1.07	0.72	1.57	1.79	2
AT2G21770	0.19	0.12	0.14	0.37	0.41	0.45
AT1G73640	0.79	0.38	0.97	2.07	2.13	1.83
AT3G03530	0.24	0.21	0.32	0.84	0.75	0.68
AT3G53650	4.23	4.32	2.99	6.17	8.4	10.05
AT4G30430	2.2	2.76	2.29	0.71	1.31	1.16
AT3G28455	3.38	2.18	2.98	5.52	6.84	9.1
AT3G09780	0.4	0.71	0.56	1.08	1.06	1.29
AT1G50560	0.6	0.74	0.78	0.34	0.21	0.12
AT1G55200	0.14	0.4	0.31	1.03	0.62	0.73
AT5G48070	2.49	1.76	2.91	0.63	1.43	0.5
AT3G01710	0.63	0.58	0.65	1.38	1.19	1.78
AT3G47210	1.07	1.27	1.26	0.61	0.46	0.5
AT2G44690	0.6	0.37	0.56	1.69	1.11	2.42
AT2G44745	1.37	0.7	1.32	3.03	2.42	2.68
AT3G53140	0.93	1.03	0.65	1.8	1.73	2.25
AT5G19060	0.56	0.49	0.44	1.11	1.27	0.98
AT1G52910	2.03	2.62	2.31	4.5	3.98	6.86
AT2G37420	0.2	0.15	0.22	0.53	0.39	0.52
AT1G75580	5.51	4.69	4.07	9.49	11.72	7.87
AT5G49800	2.16	1.51	1.1	2.96	3.86	3.81
AT4G38340	0.24	0.25	0.33	0.65	0.55	0.85
AT4G15480	0.68	1.09	0.57	1.75	1.62	1.65
AT3G53232	8.36	7.27	7.95	13.38	23.1	15.33
AT5G02370	0.47	0.48	0.77	1.2	1.13	1.41
AT1G02970	0.9	0.46	0.76	1.71	1.46	1.39
AT4G13310	1.32	1.38	1.05	0.45	0.62	0.57
AT3G05790	0.24	0.15	0.1	0.38	0.46	0.54
AT1G33840	1.35	1.71	1.78	0.61	0.72	0.33
AT4G15210	0.16	0.26	0.25	0.71	0.62	0.73
AT2G20590	0.32	0.35	0.21	0.71	1	0.84
AT1G07160	1.15	0.67	0.76	2.16	1.8	1.67
AT5G42330	0.95	0.99	0.97	1.88	2.8	2.54
AT1G26450	2.45	1.61	2.38	3.92	5.98	4.01
AT1G10550	3.15	4.48	3.9	2.39	1.2	2.25
AT5G60150	0.13	0.07	0.14	0.38	0.27	0.3
AT5G23190	0.08	0.16	0.23	0.61	0.36	0.68
AT5G26790	2.34	2.78	1.31	5.62	6.09	5.75
AT4G34380	0.98	0.62	0.75	0.39	0.22	0.19
AT3G50140	1.12	1.17	1.04	0.49	0.56	0.54
AT1G36675	2.19	1.95	2.02	0.72	0.81	1.24
AT3G11150	0.65	0.24	0.63	1.65	0.92	1.66

AT3G44050	0.15	0.09	0.12	0.33	0.36	0.28
AT1G09470	0.27	0.56	0.45	1.36	1.13	1.01
AT1G18870	0.33	0.5	0.5	0.98	0.89	1.13
AT4G24275	1	1.91	0.36	3.06	3.43	3.25
AT5G05810	0.45	0.38	0.35	1.23	0.61	1.79
AT3G45960	1.62	1.74	1.54	0.92	0.65	0.36
AT5G59720	1.21	1.58	1.73	3.69	4.13	2.65
AT3G10180	0.1	0.19	0.1	0.38	0.31	0.32
AT1G20060	0.19	0.27	0.29	0.52	0.56	0.57
AT4G22270	0.52	0.38	0.59	1.5	1.23	0.9
AT3G48550	1.89	1.93	1.54	3.25	4.7	3.59
AT1G68120	0.56	0.52	0.87	1.92	2.08	1.18
AT1G13790	0.13	0.24	0.21	0.61	0.46	0.5
AT3G18010	1.61	0.87	0.85	2.3	2.26	2.44
AT4G22860	0.38	0.12	0.26	0.75	0.66	0.74
AT5G23370	2.94	2.27	2.06	0.81	0.64	1.52
AT4G19975	1.14	1.3	1.48	2.64	1.89	3.58
AT1G23790	0.22	0.25	0.42	0.78	0.75	0.8
AT2G25940	0.51	0.45	0.76	1.15	1.33	1.23
AT2G19990	1.3	0.51	0.92	2.28	2.55	2.15
AT1G51355	1.48	2.4	2.01	4.15	3.86	5.16
AT3G20898	1.27	1.29	1.49	3.56	3.29	3.64
AT4G36110	1.76	0.73	1.38	3.54	3.86	3.18
AT3G52525	1.73	1.76	1.19	3.74	3.12	3.27
AT5G17820	1.04	0.98	1.01	0.48	0.34	0.35
AT1G16370	1.17	1.54	1.56	0.59	0.42	1.03
AT2G30540	1.68	2.11	1.67	3.3	4.69	5.09
AT3G01760	0.35	0.73	0.56	1.03	1.33	1.29
AT5G15581	2.56	2.31	2.51	5.39	5.84	6.39
AT4G19690	0.25	0.35	0.45	0.93	0.98	0.96
AT1G11125	0.43	0.53	0.61	1.4	1.05	1.67
AT1G49910	0.68	0.49	0.35	1.33	1.79	0.93
AT3G55150	0.38	0.44	0.5	0.81	0.78	1.22
AT3G24450	1.31	0.91	1.28	2.18	3.65	2.93
AT5G67110	0.93	1.13	0.95	1.64	2.63	2.41
AT1G76310	0.71	0.59	0.88	1.36	1.18	1.97
AT5G43250	1.32	3.12	2.35	5.18	4.04	5.45
AT2G47520	1.81	1.26	1.37	2.35	3.98	3.51
AT1G62440	0.53	0.29	0.56	0.18	0.18	0.14
AT1G62420	1.34	1.13	1.37	0.68	0.49	0.34
AT5G54585	1.21	1.73	1.29	3.65	4.33	2.39
AT2G22000	2.09	3.29	4.05	7.08	4.9	8.17
AT4G35170	0.73	0.81	0.69	1.56	1.56	1.69

AT3G10200	0.39	0.16	0.29	0.75	0.57	0.74
AT5G22930	0.82	1.12	0.98	2.3	1.76	2.19
AT4G33610	0.7	2.65	1.4	3.9	3.36	4.65
AT4G35620	0.45	0.36	0.63	1.2	1.01	1.03
AT5G52020	1.29	1.65	1.47	0.56	0.8	0.34
AT5G02420	1.39	1.32	1.25	3.21	3.09	3.15
AT3G07970	0.21	0.41	0.33	0.88	0.71	0.79
AT4G02700	0.36	0.24	0.28	0.55	0.63	0.81
AT5G51920	1.01	0.78	0.95	0.54	0.38	0.44
AT5G37690	0.65	0.64	0.51	1.86	1.18	1.07
AT5G02520	0.26	0.21	0.25	0.73	0.38	0.69
AT1G31750	2.8	2.74	3.78	1.64	1.06	1.97
AT3G23930	0.72	0.45	1	1.37	1.66	2.33
AT1G52245	2.19	1.75	1.9	4.6	5.25	3.51
AT3G01330	0.46	0.5	0.25	1.09	0.7	1.25
AT1G30135	3.15	4.75	4.45	1.96	1.77	2.41
AT5G06930	0.29	0.21	0.22	0.74	0.37	0.61
AT1G67260	0.25	0.35	0.35	0.44	0.99	1.22
AT2G14247	5.99	5.34	4.01	3.27	1.58	1.41
AT1G80060	1.15	0.92	0.65	2.06	1.61	2.08
AT3G46070	2.23	3.59	2.64	1.13	1.37	1.39
AT1G56240	1.66	2.02	2.95	0.96	1.04	1.28
AT4G32780	0.29	0.41	0.33	0.97	0.82	0.69
AT5G38000	0.47	0.1	0.42	1.06	0.59	1.08
AT1G33430	0.34	0.22	0.54	0.92	0.88	0.86
AT3G16390	0.91	0.82	0.85	0.41	0.37	0.41
AT3G47460	0.2	0.26	0.34	0.47	0.44	0.76
AT1G58170	1.55	1.03	1.46	3.13	3.01	2.22
AT3G14850	0.58	0.86	0.71	2.49	1.05	1.47
AT4G34790	0.64	1.64	2.07	2.97	3.14	4.66
AT4G25350	0.26	0.36	0.3	0.65	0.71	0.55
AT5G59340	1.5	1.76	1.94	0.62	0.95	0.97
AT3G27940	2.32	3.4	2.23	1.05	1.01	1.44
AT5G19040	1.12	1.31	1.26	0.64	0.66	0.43
AT1G04580	0.19	0.12	0.19	0.33	0.35	0.37
AT1G42980	0.65	1.19	1.12	0.43	0.05	0.58
AT5G24080	0.26	0.25	0.24	0.46	0.59	0.49
AT5G37670	2.84	4.35	3.41	1.41	1.81	1.96
AT1G79760	0.43	0.96	0.57	1.72	1.31	1.24
AT4G22212	2.98	4.88	4.27	1.64	1.58	2.14
AT4G24150	0.39	0.51	0.38	1.1	0.66	0.95
AT2G37260	0.75	0.27	0.44	1.43	0.98	0.92
AT1G18980	1.04	0.93	0.98	1.54	1.97	2.59

AT3G46680	0.41	0.23	0.48	0.75	1.07	0.71
AT2G30660	0.46	0.3	0.74	0.9	1.11	1.3
AT1G08860	0.75	0.76	0.76	0.58	0.24	0.3
AT3G45430	0.21	0.28	0.31	0.67	0.44	0.64
AT1G62290	0.42	0.29	0.29	0.73	0.94	0.54
AT4G17280	0.6	0.54	0.49	1.12	0.73	1.73
AT4G34420	0.34	0.37	0.42	0.57	1.14	1.02
AT3G47200	0.8	0.75	0.78	0.61	0.23	0.23
AT5G46140	1.03	1.19	1.15	0.5	0.59	0.44
AT5G16490	0.82	1.41	1.41	2.31	3.14	2.16
AT2G39880	0.69	0.66	0.42	1.89	0.8	1.21
AT1G16070	0.41	0.21	0.34	0.42	1.08	0.83
AT5G23600	0.98	1.8	1.61	0.76	0.44	0.75
AT1G72140	0.37	0.3	0.34	0.93	0.39	0.86
AT5G36790	9.51	31.28	85.01	30.51	7.68	7.79
AT4G26288	2.29	1.72	2.61	0.83	1.2	1.32
AT1G59860	0.74	1.15	1.22	2.38	2.7	1.32
AT1G80165	2.56	2.13	2.26	2.7	4.98	6.62
AT1G55980	0.65	0.86	0.36	0.64	2.48	0.97
AT1G55335	1.5	3.98	1.06	4.41	5.66	3.09
